# Characterization of MORN2 stability and regulatory function in LC3-associated phagocytosis in macrophages

**DOI:** 10.1242/bio.051029

**Published:** 2020-06-23

**Authors:** Maya Morita, Mayu Kajiye, Chiye Sakurai, Shuichi Kubo, Miki Takahashi, Daiki Kinoshita, Naohiro Hori, Kiyotaka Hatsuzawa

**Affiliations:** Division of Molecular Biology, School of Life Sciences, Faculty of Medicine, Tottori University, Yonago, Tottori 683-8503, Japan

**Keywords:** LC3-associated phagocytosis, Membrane trafficking, MORN2, Non-canonical autophagy, Phagosome maturation, SNARE protein

## Abstract

Microtubule-associated protein A1/B1-light chain 3 (LC3)-associated phagocytosis (LAP) is a type of non-canonical autophagy that regulates phagosome maturation in macrophages. However, the role and regulatory mechanism of LAP remain largely unknown. Recently, the membrane occupation and recognition nexus repeat-containing-2 (MORN2) was identified as a key component of LAP for the efficient formation of LC3-recruiting phagosomes. To characterize MORN2 and elucidate its function in LAP, we established a MORN2-overexpressing macrophage line. At a steady state, MORN2 was partially cleaved by the ubiquitin-proteasome system. MORN2 overexpression promoted not only LC3-II production but also LAP phagosome (LAPosome) acidification during *Escherichia coli* uptake. Furthermore, the formation of LAPosomes containing the yeast cell wall component zymosan was enhanced in MORN2-overexpressing cells and depended on reactive oxygen species (ROS). Finally, MORN2-mediated LAP was regulated by plasma membrane-localized soluble *N*-ethylmaleimide-sensitive factor attachment protein receptors (SNAREs) such as SNAP-23 and syntaxin 11. Taken together, these findings demonstrate that MORN2, whose expression is downregulated via proteasomal digestion, is a limiting factor for LAP, and that membrane trafficking by SNARE proteins is involved in MORN2-mediated LAP.

## Abstract (precis)

**Summary:** MORN2, whose stability is regulated by the ubiquitin-proteasome system, functions as a limiting factor for LAP via the regulation of SNAP-23 membrane trafficking for ROS production within macrophage phagosomes.

## INTRODUCTION

Phagocytosis is a type of endocytosis specific to phagocytes including macrophages. It represents a fundamental cellular process involved in immunity. During phagocytosis, large foreign particles such as pathogens or other antibody-opsonized targets and apoptotic bodies, are internalized into the cells as newly formed organelles named phagosomes, which gradually mature into phagolysosomes through a sequential interaction with endosome/lysosome-related organelles. As a result, reactive oxygen species (ROS) are produced within the phagosome, leading to an acidic environment and an accumulation of various hydrolases (Jutras and Desjardins, 2005; Underhill and Goodridge, 2012). The degree of phagosome maturation is controlled depending on the target through a process known as microtubule-associated protein 1A/1B-light chain 3 (LC3)-associated phagocytosis (LAP) (Sanjuan et al., 2007). LAP is observed during the internalization of particles that engage various receptors such as Toll-like receptors, Fc receptors, T-cell immunoglobulin and mucin domain containing 4 (TIM4), and Dectin-1. Furthermore, LAP facilitates the maturation and bactericidal activity of phagosomes (Sanjuan et al., 2007; Huang et al., 2009; Tam et al., 2014); however, in some cases LAP rather suppresses the phagosome maturation and enhances the recruitment of major histocompatibility complex (MHC) class II molecules into phagosomes, thereby promoting the presentation of target-derived peptides (Ma et al., 2012; Romao et al., 2013). This process is characterized by the recruitment of autophagy-related protein phosphatidylethanolamine (PE)-conjugated LC3 (LC3-II) to phagosomes, and the LC3-decorated phagosomes that are formed are named LAPosomes (Heckmann and Green, 2019; Upadhyay and Philips, 2019).

In contrast to canonical autophagy, LAPosomes consist of a single lipid bilayer. Furthermore, a pre-initiation complex composed of ULK1 and/or ULK2, FIP200, ATG13, and ATG101, which is essential for canonical autophagy, is not required for LAP. However, LAPosome formation requires class III phosphatidylinositol 3-kinase (PI3K)-associated proteins such as Beclin-1, VPS15, VPS34, ultraviolet radiation resistance associated gene protein (UVRAG), and Run domain Beclin-1*-*interacting and cysteine*-*rich domain*-*containing protein (Rubicon) (Martinez et al., 2015; Upadhyay and Philips, 2019). One of the unique features of LAP is that it requires ROS production by the NADPH oxidase-2 (NOX2) complex for LC3 lipidation on phagosomes; however, ROS production is dispensable for canonical autophagy (Huang et al., 2009; Martinez et al., 2015). Therefore, LAP is considered a non-canonical form of autophagy. However, the regulatory mechanism underlying its function remains to be fully elucidated.

A screen of planarian antimicrobial activity has identified an orthologous protein for mammalian membrane occupation and recognition nexus repeat-containing-2 (MORN2) (Abnave et al., 2014). In fact, MORN2 is involved in the elimination of bacteria such as *Legionella pneumophila*, *Staphylococcus aureus* and *Mycobacterium tuberculosis* through the facilitation of LAP efficiency in macrophages. MORN2-mediated LAP requires ATG5 and Beclin-1 but not ULK1 and ATG13 to form single membrane-containing LAPosomes (Abnave et al., 2014). Although MORN2-overexpressing macrophages enhance LAP efficiency (the ratio of LC3-positive phagosomes to total phagosomes) for the bacteria listed above, it is unclear whether MORN2 is essential for general ROS-dependent LAP activity. Further, the mechanism by which MORN2 regulates both LC3 recruitment to phagosomes and the phagosomal environment responsible for phagosome maturation remains unclear. Since LAP efficiency is very low in some cases (Huang et al., 2009; Matte et al., 2016), it is difficult to accurately analyze the LAP mechanism. Therefore, established MORN2-overexpressing macrophage lines may be an impactful tool for the elucidation of LAP function and regulatory mechanisms.

LAPosome formation and maturation may be regulated by membrane traffic from endocytic organelles, lysosomes and lysosome-related organelles. During membrane traffic, soluble *N*-ethylmaleimide-sensitive factor attachment protein receptors (SNAREs) play a key role in the docking and subsequent fusion of two membranes (Jahn and Scheller, 2006; Hong and Lev, 2014). Indeed, *Leishmania major* inhibits the SNARE protein vesicle-associated membrane protein 8 (VAMP8), which mediates the recruitment of NOX2 complexes to the phagosomes by cleavage using its metalloprotease GP63, resulting in halted LAPosome formation in macrophages (Matte et al., 2016). In epithelial cells, VAMP3 plays a role in the formation of single-membrane LC3-positive vacuoles containing *Yersinia pseudotuberculosis* (Ligeon et al., 2014). In contrast, we have previously demonstrated that SNAP-23, a plasma membrane-localized SNARE, regulates phagosome formation and maturation by switching its phosphorylation at Ser95 in macrophages (Sakurai et al., 2012, 2018). However, the involvement of SNAP-23 in LAP and the membrane fusion leading to LAPosome formation and maturation remain to be addressed. In this study, we investigated MORN2 stability and SNAP-23 function during LAP using macrophages stably overexpressing MORN2. The findings demonstrate that MORN2 stability at steady state is regulated by the centrosome-associated proteasome, a part of the ubiquitin-proteasome system (Wigley et al., 1999; Fabunmi et al., 2000; Vora and Phillips, 2016), and that MORN2 regulates LAPosome formation by enhancing SNAP-23 localization onto phagosomes in macrophages.

## RESULTS

### At steady state, MORN2 is partially cleaved by the ubiquitin-proteasome system

**Fig. 1.**
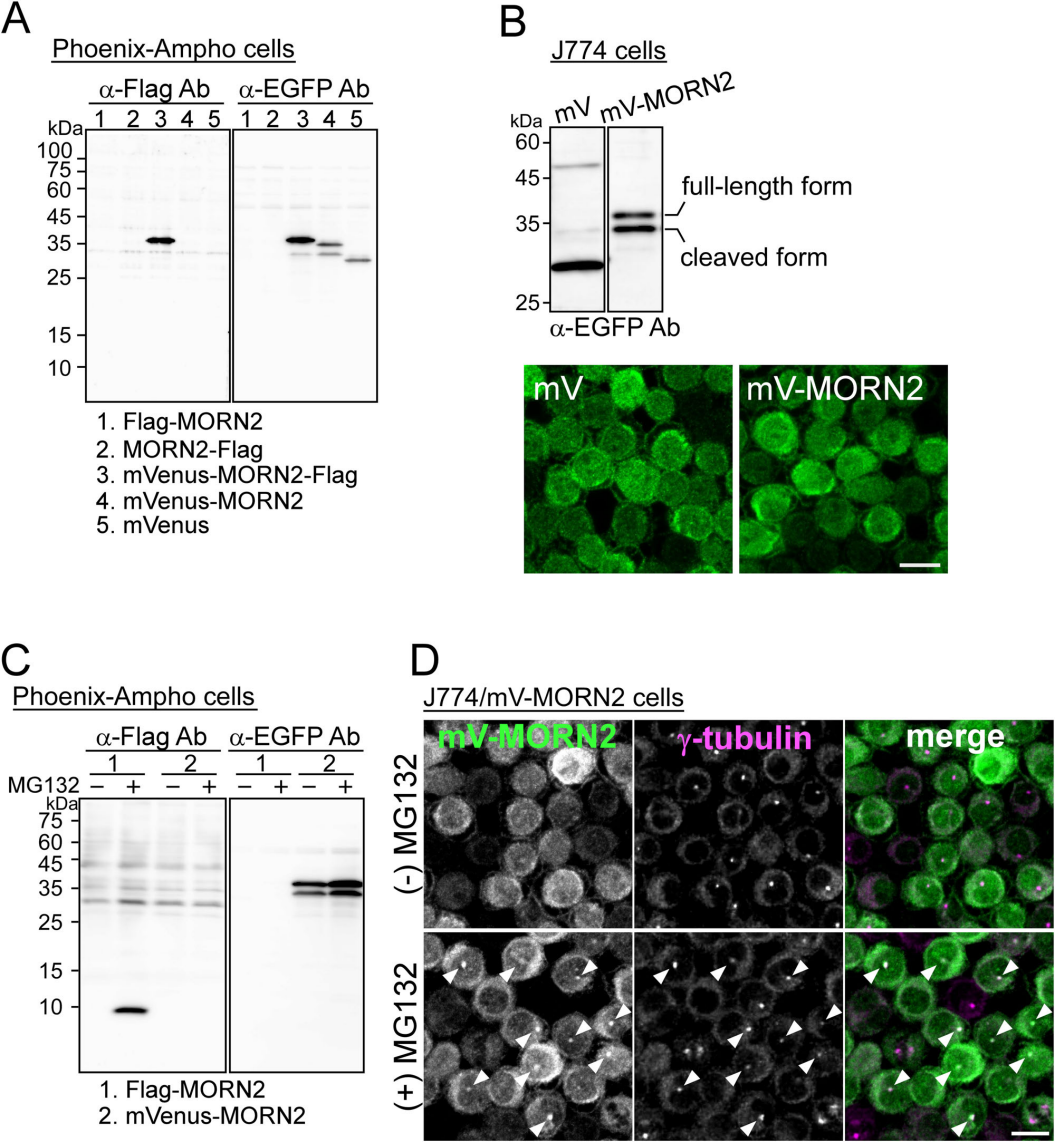
**Overexpressed MORN2 is partially cleaved near the centrosome by the ubiquitin-proteasome system.** (A) Total protein lysates from Phoenix-Ampho cells transiently expressing one of the plasmids numbered 1–5 were analyzed by WB using the indicated antibodies. (B) Total lysates from J774 cells stably expressing mVenus (mV) and mV-MORN2 were analyzed by WB using anti-EGFP antibodies (upper panel). The cells were fixed, stained with anti-EGFP antibodies and subsequently labeled with fluorescent dye-conjugated goat anti-rabbit secondary antibodies (lower panel). (C) Phoenix-Ampho cells transiently expressing Flag-MORN2 and mV-MORN2 were incubated in the presence or absence of MG132 (5 μM) for 5 h. Each total cell lysate was analyzed by WB using the indicated antibodies. (D) J774/mV-MORN2 cells incubated under the same conditions as in (C) were fixed, stained with anti-EGFP and anti-γ-tubulin antibodies, and subsequently labeled with fluorescent dye-conjugated secondary antibodies. White arrowheads indicate the co-localization of both signals. Scale bar: 10 μm.

LAP is not observed in all phagosomes, and its efficiency in macrophages is as low as 20–30% (Huang et al., 2009; Matte et al., 2016). To establish an efficient system for LAP monitoring and elucidate its regulatory mechanisms, we focused on and used a LAP-related protein, MORN2, which consists of 79 amino acid residues containing two MORN motifs (Choi et al., 2010; Abnave et al., 2014). MORN2 constructs tagged with Flag and mVenus were transiently expressed in Phoenix-Ampho cells, and protein levels were determined by western blotting (WB). As shown in Fig. 1A, only the expression of mVenus-MORN2-Flag was detected by an anti-Flag antibody (left panel), whereas an anti-EGFP antibody detected not only the full-length form of mVenus-MORN2-Flag and mVenus (mV)-MORN2 but also the 34-kDa C-terminal deletion forms (right panel). mV-MORN2 stably overexpressed in the murine macrophage-like cell line J774 was also detected by WB with an anti-EGFP antibody as two protein signals with a molecular weight of 37 kDa and 34 kDa, respectively (Fig. 1B). These results indicate that MORN2 was partially cleaved near its center region. Since mV-MORN2 was localized throughout the cytoplasm of J774 cells (Fig. 1B), proteolytic cleavage was apparently due to the ubiquitin-proteasome system. To clarify this possibility, we examined the effect of the proteasome inhibitor (MG132) on the expression of MORN2 constructs in Phoenix-Ampho cells. In the presence of MG132, an increased expression of Flag-MORN2 and mV-MORN2 full-length and truncated forms was detected (Fig. 1C). Interestingly, mV-MORN2 in J774 cells was partially co-localized with γ-tubulin at the centrosome, one of the active sites of the proteasome associated with regulatory proteins after 5 h MG132 treatment (Fig. 1D). These data suggest that MORN2 stability is regulated by the centrosome-associated proteasome (Wigley et al., 1999; Fabunmi et al., 2000; Vora and Phillips, 2016) in steady-state macrophages.

### MORN2 is not ubiquitylated with lysine residues for its proteasomal digestion

**Fig. 2.**
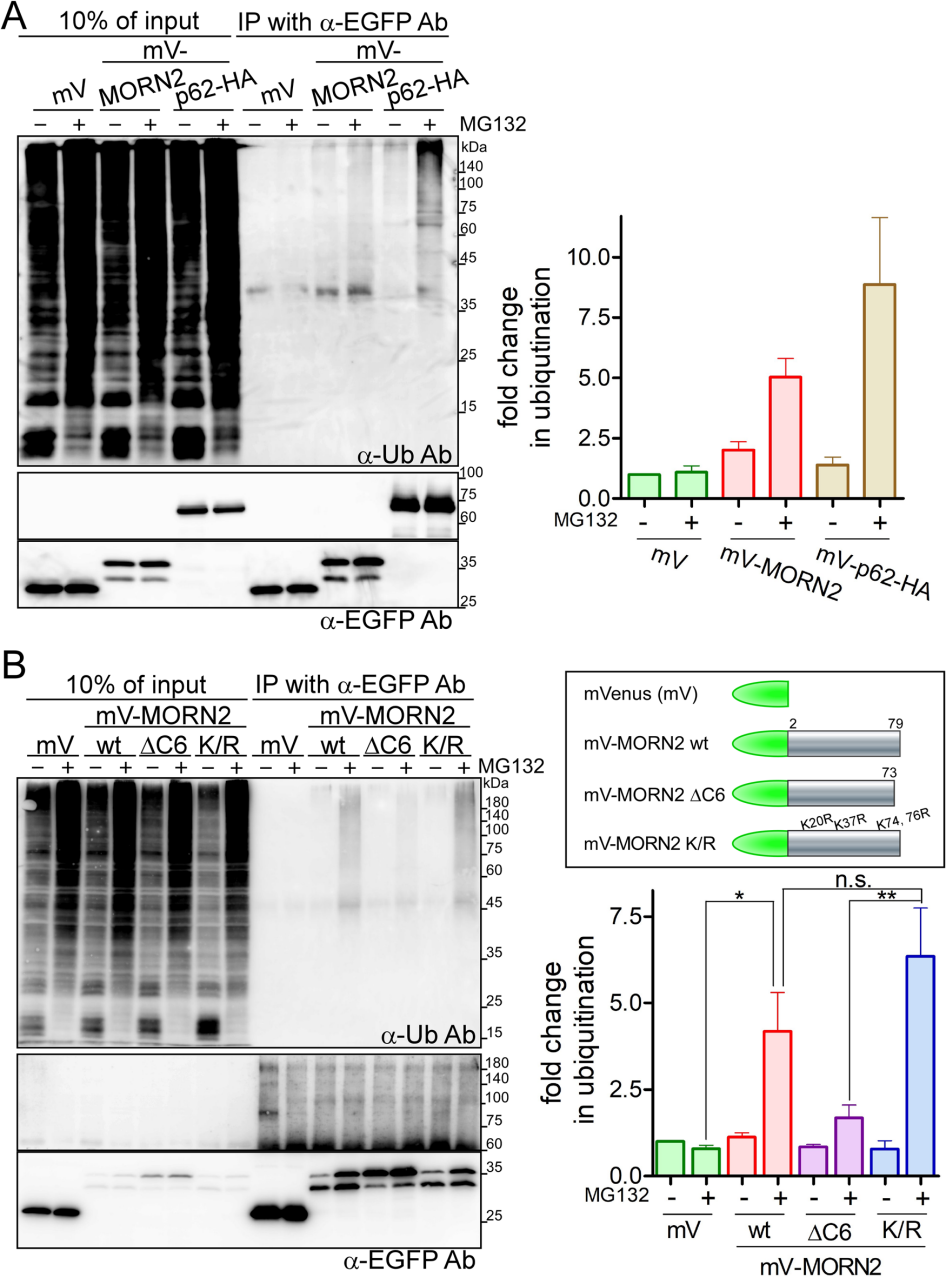
**MORN2 proteasomal cleavage depends on the C-terminal 6 amino acid residues but not on the lysine residues.** (A) Phoenix-Ampho cells transiently transfected with mVenus (mV) mV-MORN2, and mV-p62-HA were incubated in the presence or absence of MG132 (5 μM) for 5 h. Total lysates from each cell sample were coimmunoprecipitated with anti-EGFP antibodies. The immunocomplexes were subjected to SDS-PAGE followed by WB using the indicated antibodies against ubiquitin (Ub) and EGFP. High-molecular-weight protein signals over 60 kDa in the left panel were quantified using ImageJ. The ubiquitination level of mV-expressed cells without MG132 was defined as 1.0, and the fold change of each cell was normalized to this value (right panel). Data are presented as mean±s.e.m. of three independent experiments. (B) A schematic representation of the mV-MORN2 constructs. MORN2 wt (2–79 amino acid), MORN2 ΔC6 lacking its C-terminal 6 amino acid residues, and MORN2 K/R replacing all four lysine residues (K20, K37, K74, and K76) with arginine residues were tagged with mVenus as depicted (upper right panel). J774 cells stably expressing mV, mV-MORN2 wt, mV-MORN2 ΔC6, or mV-MORN2 K/R were treated with or without MG132 (5 μM) for 5 h. The cells were lysed and immunoprecipitated with anti-EGFP antibodies. The immunocomplexes were analyzed as described in (A) (left and lower right panels). Statistical analysis was performed using one-way analysis of variance (ANOVA) with Tukey's post hoc test (*P<0.05; **P<0.01; n.s., not significant).

Next, to examine whether MORN2 is ubiquitylated for proteasomal digestion, an immunoprecipitation experiment was performed with Phoenix-Ampho cells transiently transfected with mVenus-tagged constructs in the presence or absence of MG132 (Fig. 2A). When lysates from cells transfected with the mV-MORN2 construct in the presence of MG132 were used for immunoprecipitation, an increasingly pronounced smear of high-molecular-weight proteins was detected by an anti-ubiquitin antibody, which was similar to the positive control mVenus-tagged p62-HA (mV-p62-HA), an adaptor protein for autophagy with multiple protein–protein interaction domains such as ubiquitination and ubiquitin-association (Peng et al., 2017) (Fig. 2A). MORN2 contains four lysine residues (K20, K37, K74, and K76), which do not match high confidence ubiquitination sites identified by UbPred (http://www.ubpred.org/). To investigate whether these lysine residues serve as ubiquitination sites, we constructed two mutants, mV-MORN2 ΔC6 (C-terminal 6 amino acid residues deletion) and mV-MORN2 K/R (with all lysine residues replaced with arginine residues) (Fig. 2B, right panel). Lysates from J774 cells stably expressing these constructs were subjected to the same experiment, as the one presented in Fig. 2A. Remarkably, a ubiquitylated smeared band was observed in the K/R mutant treated with MG132 but not in the ΔC6 mutant (Fig. 2B, upper left panel). Indeed, mV-MORN2 ΔC6 showed considerable resistance against partial digestion compared with that of wild type (wt) and K/R (Fig. 2B, lower left panel). These data suggest that MORN2 lysine residues are not involved in its ubiquitination, and that the C-terminal 6 amino acid residues may be important for the structural formation of MORN2 and its ubiquitination or interaction with ubiquitylated proteins. The ubiquitination of amino acid residues other than lysine residues in MORN2 or the possible interaction between MORN2 and other ubiquitylated proteins remains unknown.

### MORN2 overexpression enhances the phagosomal acidification and LC3-II production during *E. coli* uptake in macrophages

**Fig. 3.**
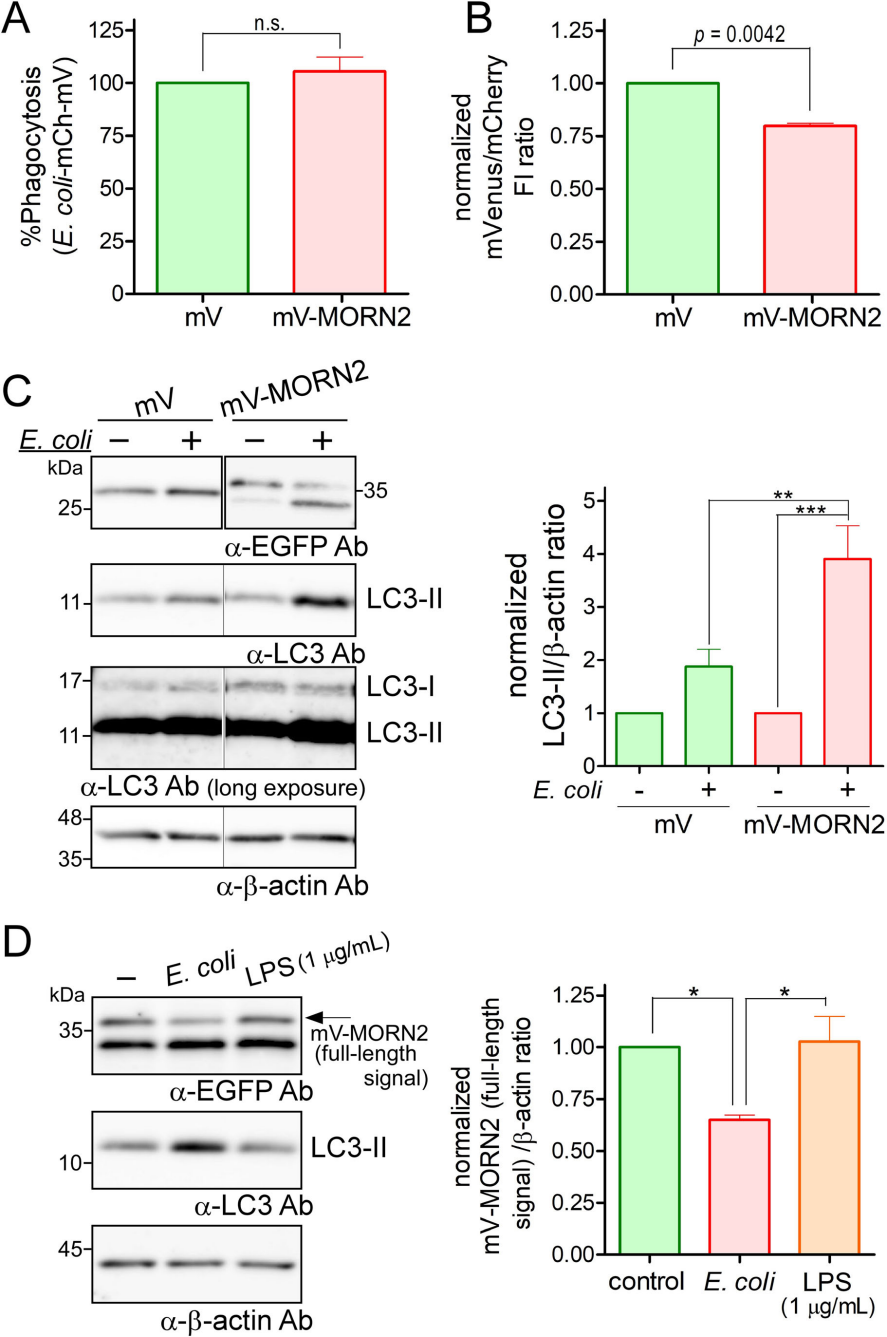
**mVenus-MORN2 overexpression enhances phagosomal acidification and LC3-II production during *E. coli* uptake.** (A) J774 cells stably expressing mV and mV-MORN2 were incubated with *E. coli*-mCherry-mVenus particles. Phagocytosis efficiency of the particles in the indicated cell types was determined by mCherry fluorescence. In each experiment, the fluorescence signal obtained with control mV cells was defined as 100% and the fluorescence derived from mV-MORN2 cells was normalized to this value. (B) Simultaneously, the phagosome maturation efficiency of each cell type was estimated using the mVenus/mCherry fluorescence intensity (FI) ratio. In each experiment, the FI ratio of the control mV cells was defined as 1.0 and the FI ratio of the mV-MORN2 cells was normalized to this value. An FI ratio <1.0 indicated enhanced phagosome maturation (acidification) compared with that of control cells (Morita et al., 2017). Data are presented as mean±s.e.m. of three independent experiments (A and B). (C) J774/mV and J774/mV-MORN2 cells were incubated with or without *E. coli* (OD600. 1.0 units) for 1 h. Each cell lysate was subjected to SDS-PAGE and WB to determine the levels of LC3-II production. β-actin expression in the same lysates was assessed by WB and served as a loading control (left panel). Protein signals in the left panel were quantified using ImageJ. LC3-II level was expressed as the ratio of LC3-II to β-actin signal intensity, which was then normalized to that of the cells incubated without *E. coli* set to 1.0 (right panel). Data are presented as mean±s.e.m. of six independent experiments. (D) Lysates from J774/mV-MORN2 cells incubated with or without *E. coli* and LPS for 1 h were analyzed by WB using the indicated antibodies (left panel). Protein signals were quantified using ImageJ. The value of the mV-MORN2 full-length signal divided by that of the total mV-MORN2 signal was expressed as a ratio to the β-actin signal. In each experiment, the intensity ratio of the full-length signal from control cells (−) was defined as 1.0, and the ratio of the full-length signal from cells incubated with *E. coli* or LPS was normalized to this value (right panel). Data are presented as mean±s.e.m. of three independent experiments. Statistical analysis was performed using a two-tailed, paired Student's *t*-test (A and B) and using one-way ANOVA with Tukey's post hoc test (C and D); *P<0.05; **P<0.01; ***P<0.001; n.s., not significant.

As the aim of J774/mV-MORN2 cell generation was to elucidate the regulatory mechanisms underlying LAP, we analyzed the phagocytic ability of the cells in several ways. Consistent with previous results for bacteria such as *Staphylococcus aureus* and *Mycobacterium tuberculosis* H37Rv (Abnave et al., 2014), mV-MORN2 overexpression in J774 cells did not affect the phagocytosis efficiency for *E. coli* (Fig. 3A). Using *E. coli* expressing GST-mCherry-mVenus (mVenus but not mCherry fluorescence decreases in an acidic environment) to detect phagosomal acidification (Morita et al., 2017), J774/mV-MORN2 cells exhibited significantly enhanced phagosome maturation (acidification) efficiency compared with that of the control cells expressing mVenus (Fig. 3B). In addition to acidification, phagosomal degradation activity was also enhanced in J774/mV-MORN2 cells (Fig. S1).

The LC3 localization on the phagosome containing *E. coli* was not clearly visible in the immunofluorescence assay, probably because the low amount of LC3-II on the phagosome was indistinguishable from the cytoplasmic background. However, enhanced LC3-II production was detected in J774/mV-MORN2 cells when compared with that in control cells using WB during *E. coli* uptake (Fig. 3C). This is probably because the amount of LC3-II on the phagosome was low but the number of phagosomes was high. This LC3-II production apparently enhances the recruitment of lipidated (PE-conjugated) LC3 to phagosomes containing *E. coli* in J774/mV-MORN2 cells. Since the partial cleavage of mV-MORN2 was unexpectedly promoted during *E. coli* uptake (Fig. 3C), we examined the involvement of the Toll-like receptor 4 (TLR4) signaling pathway in LAP regulation. As shown in Fig. 3D, stimulation of lipopolysaccharide (LPS), one of the TLR4 ligands, did not affect the cleavage of mV-MORN2 and LC3-II production, indicating that this may occur concomitantly with LAPosome formation during *E. coli* uptake.

### MORN2 overexpression promotes LC3 recruitment onto zymosan-containing phagosomes in a manner dependent on ROS production in macrophages

**Fig. 4.**
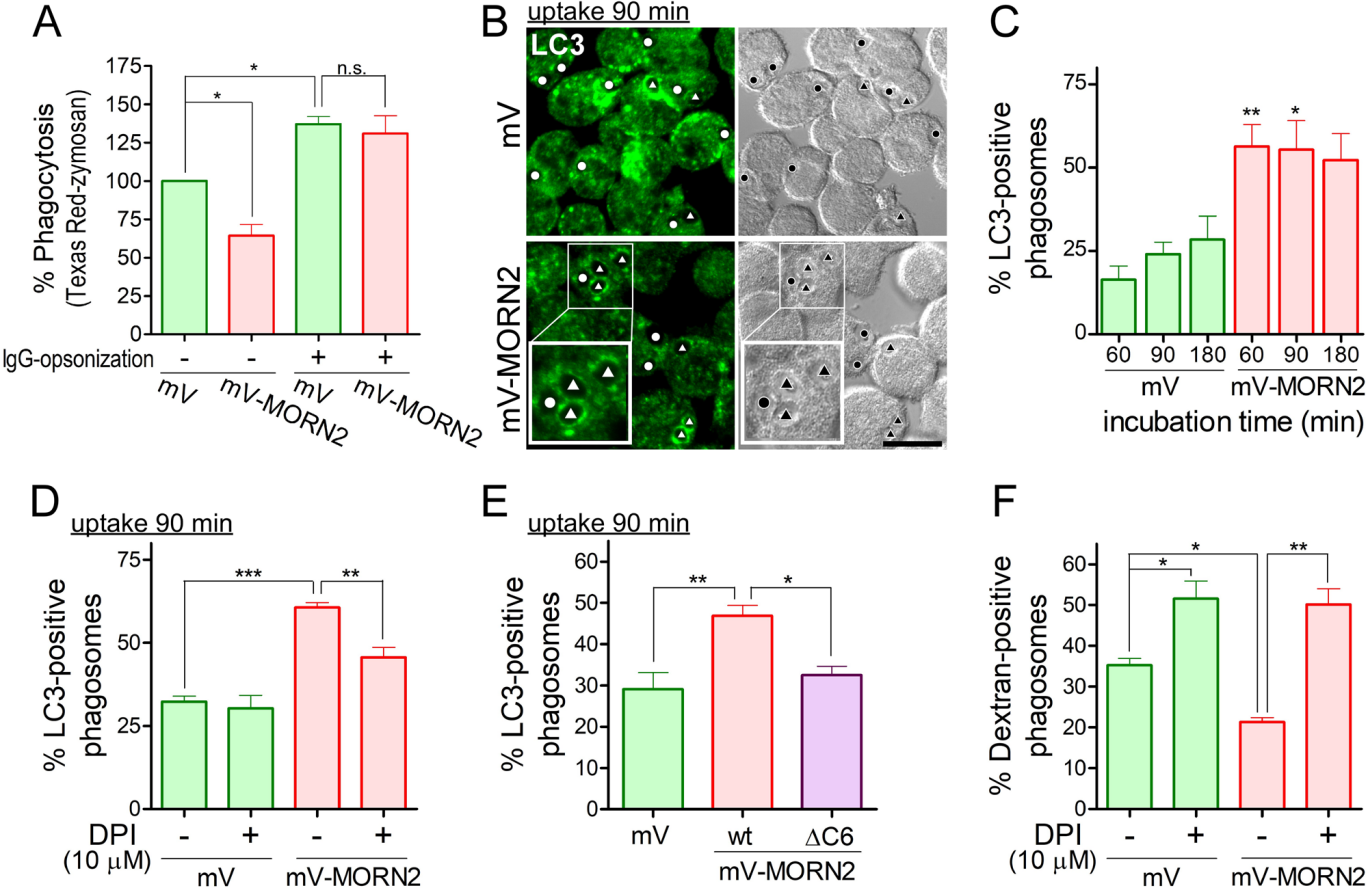
**mVenus-MORN2 overexpression promotes zymosan-containing LAPosome formation, which depends on ROS production by the NOX2 complex.** (A) J774/mV and J774/mV-MORN2 cells were incubated with Texas Red-zymosan (−) and IgG-opsonized Texas Red-zymosan (+) particles for 1 h, and then phagocytosis efficiency (%) was measured as described in Materials and Methods. Fluorescence values were normalized to the value obtained from mV cells treated with Texas Red-zymosan (−), which was arbitrarily defined as 100%. Data are presented as mean±s.e.m. of three independent experiments. (B) J774/mV and J774/mV-MORN2 cells were incubated with zymosan particles for 90 min, fixed, stained with anti-LC3 antibodies, and labeled with fluorescent dye-conjugated secondary antibodies. Triangles and circles indicate LC3 signal-positive phagosomes (LAPosomes) and no signal phagosomes, respectively. Scale bar: 10 μm. (C) J774/mV and J774/mV-MORN2 cells were incubated with zymosan particles for the indicated time periods. The cells were fixed and stained as described in B. The percentage of LC3-positive phagosomes (more than 30 individual phagosomes from at least 20 different cells were measured in each experiment) was calculated. Data are presented as mean±s.e.m. of four independent experiments. (D) J774/mV and J774/mV-MORN2 cells were incubated with zymosan particles for 90 min in the presence or absence of DPI. As in C, the cells were prepared, and phagosomes were counted. Next, the percentage of LC3-positive phagosomes was calculated. Data are presented as mean±s.e.m. of five to six independent experiments. (E) J774/mV, J774/mV-MORN2 wt, and ΔC6 mutant cells were incubated with zymosan particles for 90 min. As in C, the cells were prepared, and phagosomes were counted. Next, the percentage of LC3-positive phagosomes was calculated. Data are presented as mean±s.e.m. of four independent experiments. (F) J774/mV and J774/mV-MORN2 cells were analyzed for the fusion efficiency of phagosomes and lysosomes stained with CF640R-dextran. Cells loaded with CF640R-dextran were washed and further incubated in fresh dextran-free growth medium for 5 h. These cells were then incubated with zymosan particles in the presence or absence of DPI, and images of CF640R-dextran-positive phagosomes and unlabeled phagosomes were captured using a confocal laser-scanning microscope. The results are expressed as the percentage of CF640-dextran-positive phagosomes (more than 40 individual phagosomes for each experiment). Data are presented as mean±s.e.m. of three independent experiments. Statistical analysis was performed using one-way ANOVA with Tukey's post hoc test (*P<0.05; **P<0.01; ***P<0.001).

We further investigated the LAP efficiency in J774/mV-MORN2 cells against other relatively large targets such as zymosan (yeast cell walls). The mV-MORN2 overexpression significantly and efficiently reduced the phagocytosis of Texas Red-zymosan compared with that of control cells but not the phagocytosis of zymosan opsonized with IgG (Fig. 4A) without affecting the association of zymosan with the cells (Fig. S2). These results indicate that the effect of MORN2 overexpression on phagocytosis depends on target-specific receptors rather than on size; however, the function of MORN2 in phagocytosis remains unknown. For LAP efficiency, because no enhancement of LC3-II production during zymosan uptake was detected in J774/mV-MORN2 cells by WB, possibly due to the lower efficiency of phagocytosis than that of *E. coli*, we counted the number of LAPosomes (Fig. 4B, white triangle), which are LC3-positive phagosomes, visualized with an anti-LC3 antibody. During over 60 min of incubation for engulfment, more than 50% of all phagosomes detected in J774/mV-MORN2 cells were LC3-positive (Fig. 4C). The number of LC3-positive phagosomes in J774/mV-MORN2 cells was significantly reduced in the presence of the NOX2-selective inhibitor diphenyleneiodonium (DPI), suggesting that MORN2-mediated LAPosome formation is dependent on ROS production (Fig. 4D). Next, we examined the function of mV-MORN2 ΔC6 in LAPosome formation. As shown in Fig. 4E, stable overexpression of the mV-MORN2 ΔC6 mutant did not cause a significant enhancement of LAPosome formation compared with that of mV-MORN2 wt; furthermore, the overexpression of each mV-MORN2 protein did not affect the expression of other proteins such as LC3-II (Fig. S3). These results strongly suggest that the effect of mV-MORN2 overexpression on LAP efficiency is due to the intrinsic function of MORN2 and depends on its short C-terminal region (6 aa).

In contrast to *E. coli* (Fig. 3B), phagosomes containing zymosan in J774/mV-MORN2 cells showed significantly reduced maturation, which was estimated by counting phagosomes fused with lysosomes loaded with CF640R-dextran (dextran-positive phagosomes) (Fig. 4F). This reduced maturation efficiency was restored to the same level as in control cells by DPI treatment (Fig. 4F). The results suggest that the maturation efficiency of phagosomes in J774/mV-MORN2 cells depends on the targets and NOX2-induced ROS production.

**Fig. 5.**
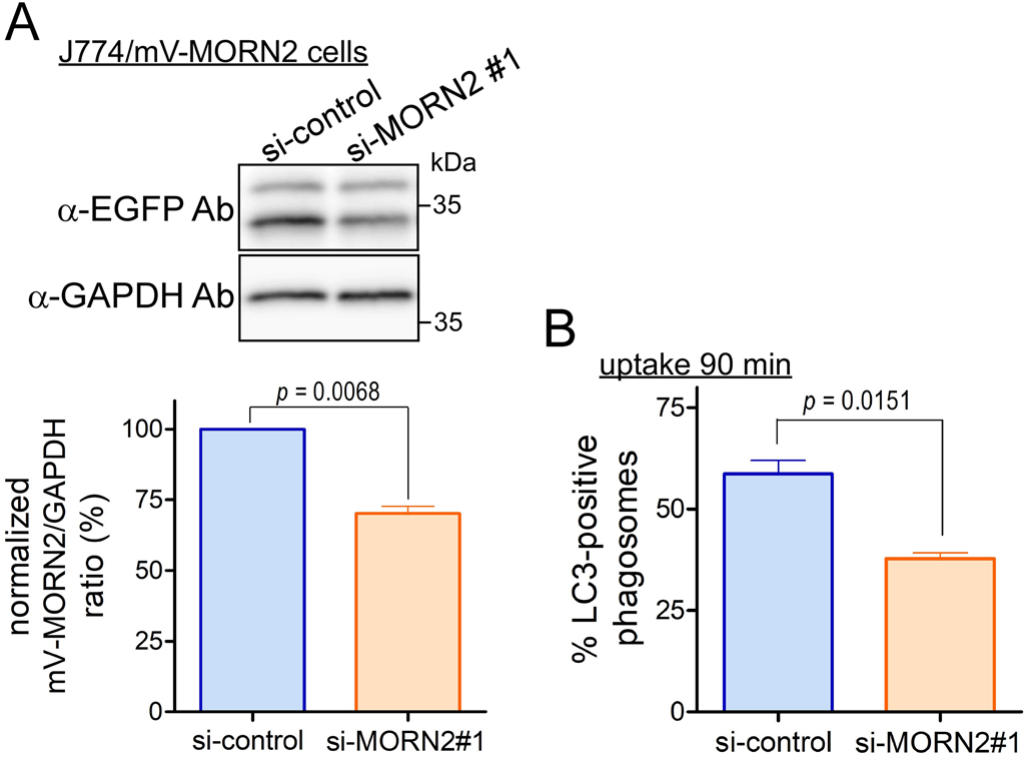
**The LAPosome formation activity of J774/mVenus-MORN2 cells is reduced by transfection with MORN2 siRNA.** (A) Total protein lysates from J774/mV-MORN2 cells at 96 h after transfection with MORN2 siRNA #1 were analyzed by WB using the indicated antibodies. Protein signals were quantified using ImageJ. The total value of mV-MORN2 (full-length and cleaved forms) was expressed as a ratio of the mV-MORN2 to the GAPDH internal control value in both control and MORN2 siRNA#1 cells and then normalized to the respective control value defined as 100%. Data are presented as mean±s.e.m. of three independent experiments. (B) At 96 h after transfection with the indicated siRNAs, J774/mV-MORN2 cells were incubated with zymosan particles and subjected to the LAP assay as described in Fig. 4. Data are presented as mean±s.e.m. of three independent experiments. Statistical analysis was performed using a two-tailed, paired Student's *t*-test.

The effect of mV-MORN2 overexpression on LAP efficiency was further confirmed by knockdown experiments. To validate the knockdown of MORN2 siRNAs in J774 cells, *MORN2* mRNA expression was determined by RT-PCR, because it was difficult to obtain workable anti-MORN2 antibodies. Transfection with si-MORN2 #1 and #2 significantly reduced *MORN2* mRNA expression by less than 50% compared with that of control siRNA (Fig. S4). The effect of transfection with si-MORN2 #1 corresponding to the open reading frame of *MORN2* mRNA on LAP efficiency was investigated in J774/mV-MORN2 cells. The mV-MORN2 expression was decreased by approximately 30% compared with that of control siRNA (Fig. 5A), whereas the LAPosome formation efficiency was significantly reduced in mV-MORN2 cells transfected with si-MORN2 #1 (Fig. 5B).

### SNAP-23 is required for MORN2-mediated LAP through ROS production by the NOX2 complex in macrophages

**Fig. 6.**
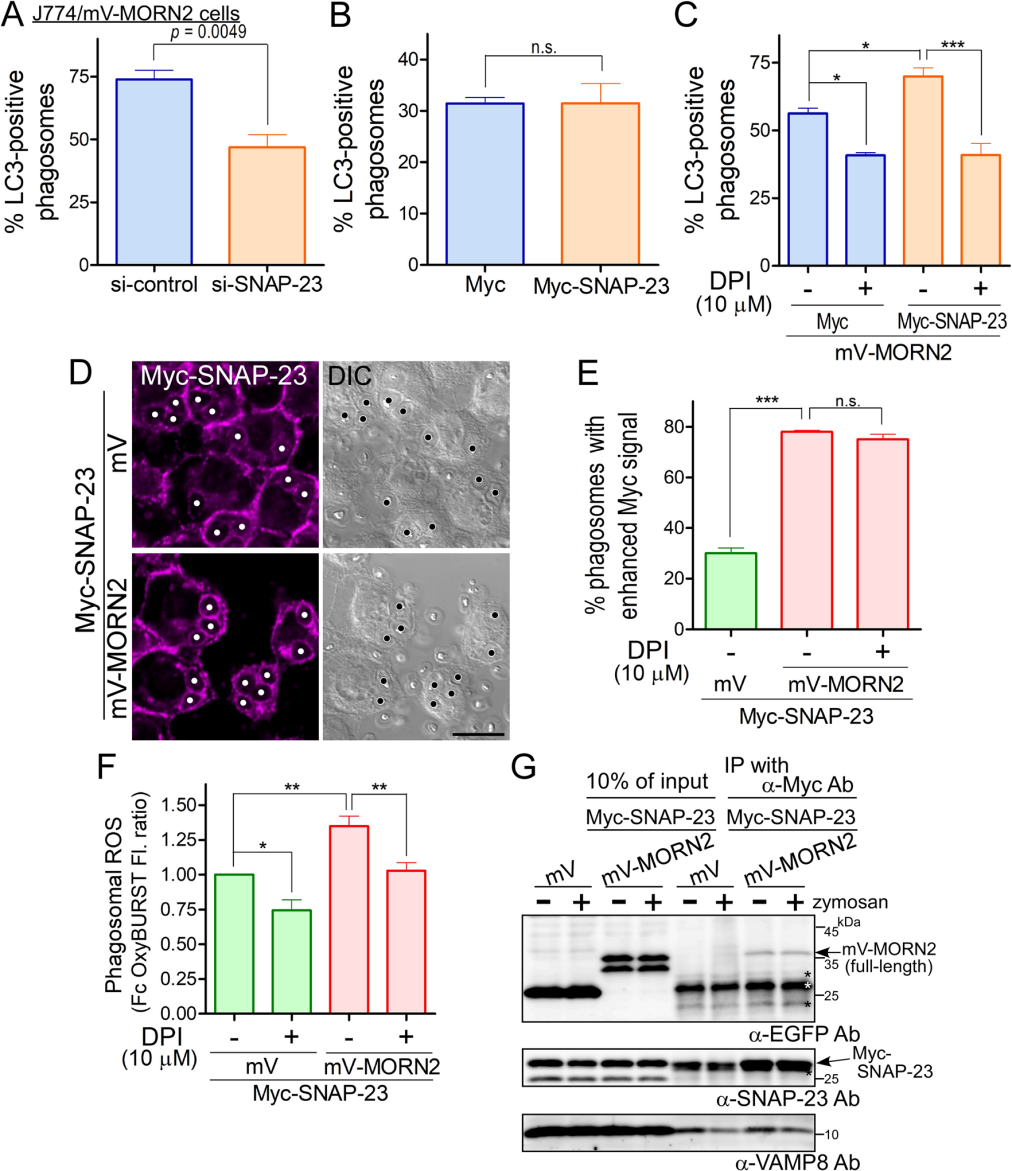
**SNAP-23 is involved in MORN2-mediated LAP through ROS production by NOX2.** (A) At 72 h after transfection with SNAP-23 siRNA, J774/mV-MORN2 cells were incubated with zymosan particles for 90 min and subjected to the LAP assay described in Fig. 4. Data are presented as mean±s.e.m. of five independent experiments. (B) J774 cells stably expressing the Myc-vector or Myc-SNAP-23 were incubated with zymosan particles for 90 min and subjected to the LAP assay described in Fig. 4. Data are presented as mean±s.e.m. of four independent experiments. (C) J774/mV-MORN2 cells stably co-expressing the Myc-vector or Myc-SNAP-23 were incubated with zymosan particles for 90 min in the presence or absence of DPI and were subjected to the LAP assay described in Fig. 4. Data are presented as mean±s.e.m. of four independent experiments. (D) J774/mV/Myc-SNAP-23 and J774/mV-MORN2/Myc-SNAP-23 cells were incubated with zymosan particles for 90 min and subjected to the immunofluorescence experiment using an anti-Myc antibody followed by fluorescent dye-conjugated secondary antibodies. Circles indicate Myc-SNAP-23-positive phagosomes. Scale bar: 10 μm. (E) J774/mV/Myc-SNAP-23 and J774/mV-MORN2/Myc-SNAP-23 cells were incubated with zymosan particles for 90 min in the presence or absence of DPI and subjected to the immunofluorescence experiment described in D. The number of phagosomes with a Myc-SNAP-23 signal (enhanced Myc signal) equal to or more intense than that of the plasma membrane signal was analyzed under a microscope. Results are expressed as the percentage of phagosomes with an enhanced Myc signal to the total phagosomes with a Myc signal (more than 40 phagosomes for each experiment). Data are presented as mean±s.e.m. of three to four independent experiments. (F) To monitor the phagosomal ROS production, J774/mV/Myc-SNAP-23 and J774/mV-MORN2/Myc-SNAP-23 cells were incubated with the Fc OxyBURST reagent for 90 min in the presence or absence of DPI, and then the fluorescence signal from the reagent was measured as described in Materials and Methods. In each experiment, the signal level from the mV/Myc-SNAP-23 cells without DPI was defined as 1.0, and the signal form each cell was normalized to this value as an FI ratio. Data are presented as mean±s.e.m. of three independent experiments. (G) Total lysates from each J774 cell co-overexpressing mVenus-tagged proteins and Myc-SNAP-23 were coimmunoprecipitated with anti-Myc antibodies and then analyzed by WB using the indicated antibodies. The asterisk indicates a signal due to the IgG light chain from IP. Statistical analysis was performed using a two-tailed, paired Student's *t*-test (A and B) and one-way ANOVA with Tukey's post hoc test (C, E and F); *P<0.05; **P<0.01; ***P<0.001; n.s., not significant.

SNAP-23 regulates not only phagosome formation and maturation but also ROS production via NOX2 complex formation on both the plasma membrane and phagosomes in macrophages (Sakurai et al., 2012). The suppression of MORN2-mediated LAP in the presence of DPI (Fig. 4D) prompted us to investigate the involvement of SNAP-23 in LAP of J774/mV-MORN2 cells. The transfection of J774/mV-MORN2 cells with SNAP-23 siRNA downregulated the expression of SNAP-23 to around 60% or less (Fig. S5), and the LAPosome formation efficiency for zymosan particles was significantly inhibited compared with that of control siRNA (Fig. 6A). Quantification of LC3-II production by WB is not suitable for the estimation of LAP efficiency, because phagosome formation efficiency changes depending on the knockdown or overexpression of SNAP-23 in macrophages (Sakurai et al., 2012). Therefore, we used zymosan particles to test the effect of SNAP-23 overexpression on LAP efficiency. Although SNAP-23 overexpression had previously been shown to enhance ROS production within phagosomes (Sakurai et al., 2012), no significant difference in LAPosome formation efficiency was observed between J774/Myc and J774/Myc-SNAP-23 cells (Fig. 6B). These data indicate that ROS production induced by SNAP-23 overexpression alone may not be sufficient to increase LAP efficiency. To clarify the relationship between MORN2 and SNAP-23 in LAP, we established J774/mV-MORN2 cells stably co-expressing the Myc-vector or Myc-SNAP-23 (Fig. S6). J774/mV-MORN2/Myc-SNAP-23 cells exhibited a significant enhancement of LAP efficiency for zymosan particles compared with that of the cells expressing Myc (Fig. 6C). Notably, this increase in LAP efficiency was remarkably suppressed in the presence of DPI to almost the same level as in control J774/mV-MORN2/Myc cells (Fig. 6C). These results suggest that MORN2 is a rate-limiting factor for LAP that promotes SNAP-23 function for phagosomal ROS production in macrophages. To test this possibility, we confirmed that Myc-SNAP-23 expression did not differ between J774/mV/Myc-SNAP-23 and J774/mV-MORN2/Myc-SNAP-23 cells (Fig. S6) and examined whether mV-MORN2 enhanced the recruitment of SNAP-23 onto phagosomes in J774/mV-MORN2/Myc-SNAP-23 cells. Using zymosan for phagocytosis, Myc-SNAP-23 was localized to numerous phagosomes (Fig. 6D, white circles), but the number of phagosomes with remarkably enhanced Myc signals was increased in J774/mV-MORN2/Myc-SNAP-23 cells regardless of the presence of DPI (Fig. 6D and E). This suggests that the MORN2-dependent enrichment of SNAP-23 onto phagosomes occurs before ROS production by the NOX2 complex. Using the Fc OxyBURST reagent (H_2_DCF-BSA immune complexes) for phagocytosis (Desai et al., 2007), the phagosomal ROS production was significantly higher in J774/mV-MORN2/Myc-SNAP-23 cells than in control J774/mV/Myc-SNAP-23 cells (Fig. 6F), whereas the phagocytosis efficiency did not differ between the two cell types (Fig. S7). Next, to clarify the interaction between MORN2 and SNAP-23 further, immunoprecipitation experiments with an anti-Myc antibody were performed using lysates of J774/mV/Myc-SNAP-23 and J774/mV-MORN2/Myc-SNAP-23 cells. Interestingly, the interaction between the mV-MORN2 full-length form and Myc-SNAP-23 was detected with or without zymosan (Fig. 6F).

**Fig. 7.**
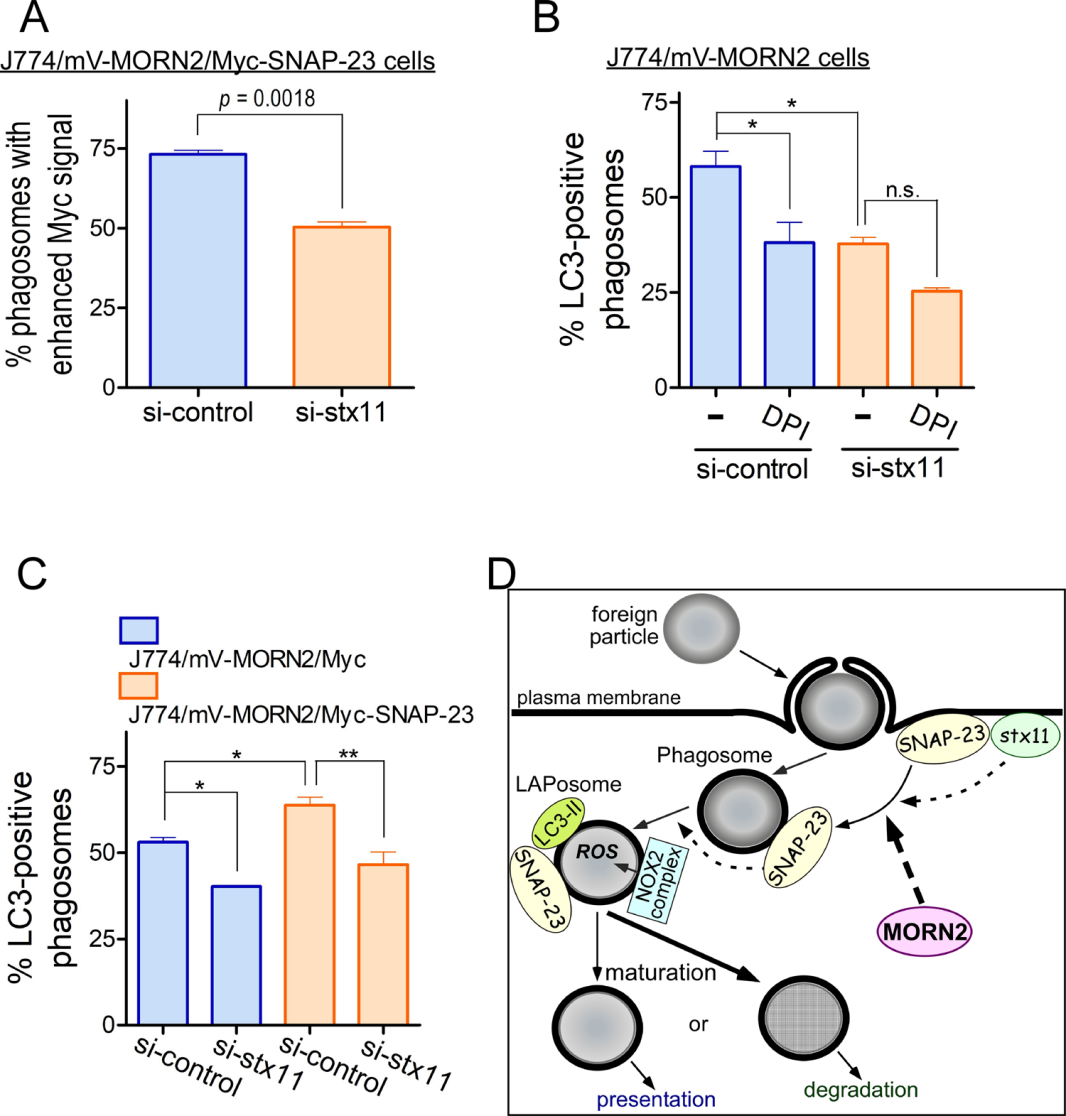
**Stx11 is an indispensable SNARE partner of SNAP-23 in MORN2-mediated LAP.** (A) At 72 h after transfection with stx11 siRNA, phagosomes with enhanced Myc signal in J774/mV-MORN2/Myc-SNAP-23 cells were analyzed as described in Fig. 6E. Data are presented as mean±s.e.m. of three independent experiments. (B and C) At 72 h after transfection with stx11 siRNA, J774/mV-MORN2 cells (B) and J774/mV-MORN2/Myc and J774/mV-MORN2/Myc-SNAP-23 cells (C) were incubated with zymosan particles for 90 min and subjected to the LAP assay described in Fig. 4. Data are presented as mean±s.e.m. of three independent experiments. Statistical analysis was performed using a two-tailed, paired Student's *t*-test (A) and one-way ANOVA with Tukey's post hoc test (B and C); *P<0.05; **P<0.01; n.s., not significant. (D) Schematic representation of MORN2-mediated LAP. During phagocytosis, MORN2 enhances the recruitment of SNAP-23, whose function is controlled by stx11, to the newly formed phagosome. SNAP-23 regulates the phagosomal ROS production by promoting NOX2 complex formation. Phagosomal ROS induce the recruitment of LC3-II onto phagosomes (LAPosome formation). LC3-II may maintain the phagosomal localization of SNAP-23 to enhance or suppress the fusion of phagosomes with endocytic compartments depending on foreign particles (see Discussion).

We had previously shown that SNAP-23 and syntaxin 11 (stx11) cooperatively regulated stimulus-dependent transport of TLR4 to the plasma membrane (Kinoshita et al., 2019). Thus, the effect of *stx11* knockdown on increased phagosomes with enhanced Myc signals was examined in J774/mV-MORN2/Myc-SNAP-23 cells. We found that *stx11* knockdown significantly reduced the number of phagosomes with enhanced Myc signals in J774/mV-MORN2/Myc-SNAP-23 cells (Fig. 7A). Importantly, *stx11* knockdown (Fig. S8) significantly reduced the LAPosome formation efficiency in J774/mV-MORN2, J774/mV-MORN2/Myc, and J774/mV-MORN2/Myc-SNAP-23 cells compared with control (Fig. 7B and C), whereas there were no significant differences in the expression of other proteins between stx11 siRNA-transfected cells (Fig. S8). Although SNAP-23 and stx11 are predominately localized on the plasma membrane of macrophages and cooperate to exert their function (Kinoshita et al., 2019), stx11 was found to be an indispensable SNARE partner of SNAP-23 even in MORN2-mediated LAP. These results indicate that MORN2 may upregulate the recruitment of SNAP-23, which is in cooperation with stx11, onto phagosomes for ROS production (probably NOX2 complex formation), leading to LAP.

## DISCUSSION

MORN2 was originally identified as a protein presumably involved in acrosome biogenesis in the testes (Choi et al., 2010); however, its function remained unknown. Recently, a screening against antibacterial activity in planarians identified MORN2 as a LAP-related protein that promotes LAPosome maturation in macrophages for killing bacteria (Abnave et al., 2014). However, the precise mechanism underlying the effect of MORN2 in the LAP reaction is poorly understood. In this study, we demonstrated that MORN2 stability is regulated by the ubiquitin-proteasome system near the centrosome and established a macrophage line overexpressing MORN2 to characterize the molecular mechanism of LAP. Using this cell line, we found that MORN2-mediated LAP is ROS-dependent, the progression of phagosome maturation depends on the target particles (or pattern-recognition receptors), and LAP is controlled by the MORN2-dependent recruitment of SNAP-23, which co-operates with stx11, onto phagosomes. This study is the first to demonstrate the involvement of membrane trafficking in MORN2-mediated LAP in macrophages.

The partial cleavage of exogenously overexpressed MORN2 was reduced by MG132-mediated inhibition of the ubiquitin-proteasome system. Accumulation of overexpressed MORN2 near the centrosome in the presence of MG132 suggests that the partial cleavage may be caused by the centrosome-associated proteasome, a part of the ubiquitin-proteasome system (Wigley et al., 1999; Fabunmi et al., 2000; Vora and Phillips, 2016). Although the centrosome-associated proteasome is involved in the regulation of several biological factors such as centrosomal factors, cell cycle regulatory factors, and cell fate determinants (Vora and Phillips, 2016), the significance of MORN2 regulation near the centrosome remains unknown. The results using a MORN2 K/R mutant, in which all four lysine residues were replaced with alanine residues, suggest that MORN2 may be ubiquitylated at an amino acid other than a lysine residue. This type of ubiquitination may occur by E3 ubiquitin ligases such as Kip1 ubiquitination-promoting complex protein 1 (KPC1), whose ubiquitination partially processes the NF-κB precursor p105 to the p50 active subunit by proteasomal activity (Kravtsova-Ivantsiv et al., 2015). Alternatively, MORN2 may be cleaved by a factor with limited proteolytic activity that is inactivated by another factor, which is degraded by the ubiquitin-proteasome system at steady state. In any case, MORN2 digestion did not result from its overexpression, yet MORN2 expression may be maintained at a low steady state until a cell emergency arises. However, our results suggest that this digestion is promoted during *E. coli* uptake (LAPosome formation) independently of TLR signaling alone. Eventually, MORN2 stability is regulated by the ubiquitin-proteasome system, but the precise molecular mechanism remains unclear.

MORN2 regulation of the efficiency of LAPosome formation and maturation is target-dependent. Uptake efficiency in J774/mV-MORN2 cells was reduced for (non-opsonized) zymosan but not affected for IgG-opsonized zymosan and *E. coli*. Similar to previous results (Abnave et al., 2014), these cells exhibited an enhanced maturation efficiency such as acidification and degradation of phagosomes containing *E. coli*. However, the fusion efficiency of zymosan-containing phagosomes with lysosomes, which is one of the indicators of phagosome maturation, was delayed in these cells. Notably, delayed LAPosome maturation leads to adequate antigen processing for MHC class II presentation (Ma et al., 2012; Romao et al., 2013). Since each of these targets is recognized by a different receptor during phagocytosis (or LAP), MORN2 may not only maintain the activity of each receptor for uptake but may also regulate LAPosome maturation downstream of each receptor. J774/mV-MORN2 cells additionally expressing Myc-SNAP-23 enhanced LAPosome formation for zymosan compared with that of cells expressing the Myc vector and J774 cells expressing Myc-SNAP-23 alone, suggesting that MORN2 regulates intracellular membrane trafficking to accelerate or decelerate several membrane fusion events during LAP (Fig. 7D).

ROS production within phagosomes is required for the early process of LAP (Huang et al., 2009; Martinez et al., 2015). Consistent with this, MORN2-mediated LAP was also inhibited by a NOX2 inhibitor. We have previously found that SNAP-23 overexpression promotes ROS production within phagosomes, indicating that SNAP-23 may mediate NOX2 complex assembly on phagosomes by vesicular trafficking (Sakurai et al., 2012). Therefore, ROS production required for LAP may be achieved by SNAP-23 recruitment onto phagosomes by MORN2. Indeed, VAMP8, one of the potential SNARE partners for SNAP-23, regulates ROS production for LAP as evidenced by the *Leishmania parasitizes*-producing metalloprotease GP63 (Matte et al., 2016). ROS within phagosomes can cause PE-lipidation of LC3 (LC3-II) onto the phagosome surface, whereas SNAP-23 interacts with the membrane through central palmitoylated cysteine residues. Maintaining the localization (or enrichment) of SNAP-23 in the LAPosome membrane may be caused by intramembrane interactions between PE from LC3 and the palmitoyl group (Ra et al., 2016), leading to the regulation of membrane fusion during subsequent LAPosome maturation. It may be possible to further enhance their interaction via the participation of stx11 that also is palmitoylated at the C-terminal cysteine residues (Hong and Lev, 2014).

LAPosome maturation depends on the targets and/or their receptors, and its progress is likely to be controlled at both extremes. Enriched SNAP-23 on LAPosomes may regulate the acceleration or deceleration of membrane fusion with endocytic and lysosome-related organelles and lysosomes. The function of SNAP-23 in the phagosome membrane is regulated by the phosphorylation of the serine residue at position 95 (Ser95), and this phosphorylated form delays the phagosome maturation during Fc receptor-mediated phagocytosis (Sakurai et al., 2018). Downstream signaling of the target-bound receptor that activates kinases and/or phosphatases may alter the phosphorylation state of SNAP-23 at Ser95 or other sites for the regulation of LAPosome maturation. Our findings suggest that MORN2 promotes and regulates LAPosome formation and maturation by recruiting SNAP-23 onto phagosomes in macrophages. In future studies, to elucidate the molecular mechanism underlying LAP in macrophages, we aim to explore the precise regulation of MORN2 for SNAP-23 recruitment onto phagosomes, the interaction of LC3-II and SNAP-23 in the membrane, and the regulatory mechanism of SNAP-23 target-dependent phosphorylation.

## MATERIALS AND METHODS

### Antibodies

Rabbit polyclonal antibodies against the enhanced green fluorescent protein (EGFP) and syntaxin 11 prepared as described previously (Sakurai et al., 2012) were used at 1:1000-1:5000 dilution for WB, at 1:200-1:1000 for immunostaining, and at 1:400 for immunoprecipitation. The anti-Myc antibody raised from murine 9E10 hybridoma (ATCC, Manassas, VA) was used at 1:1000 for WB and at 1:100 for immunostaining. The remaining antibodies were obtained from the following commercial sources: rabbit anti-ubiquitin (1:500 for WB, P4D1) and rabbit anti-p22*^phox^* (1:1000 for WB, FL-195) from Santa Cruz Biotechnology (Dallas, TX, USA); mouse anti-gp91*^phox^* (1:1000 for WB, #611414, BD Transduction Laboratories, San Jose, CA, USA); mouse anit-LC3 (1:2000 for WB and 1:1000 for immunostaining, L8918), rabbit anti-SNAP-23 (1:10,000 for WB, S2194 [TS-19]), mouse anti-Flag (1:2000 for WB, F3165), mouse anti-γ-tubulin (1:250 for IF, T6557 [GTU-88]), and mouse anti-β-actin (1:20,000 for WB, A1978 [AC-15]) from Sigma-Aldrich (St Louis, MO, USA); mouse anti-glyceraldehyde-3-phosphate dehydrogenase (GAPDH) (1:15,000 for WB, AM4300 [6C5]) from Ambion (Austin, TX, USA). Secondary antibodies were Alexa 488-conjigated goat anti-rabbit IgG (1:1000, A-32731), Alexa 555-conjugated goat anti-mouse IgG (1:1000, A-21424) from Thermo Fisher Scientific (Osaka, Japan), Alexa 405-conjugated donkey anti-rabbit IgG (1:500, ab175651) from Abcam (Cambridge, UK), horseradish peroxidase (HRP)-conjugated anti-rabbit IgG (1:20,000, #611-1323) from Rockland Immunochemicals (Gilbertsville, PA, USA), or HRP-conjugated anti-mouse IgG (1:15,000, W402B) from Promega (Madison, WI, USA).

### Cell culture

J774 murine macrophage-like cells were obtained from Riken Cell Bank (Tsukuba, Japan) and cultured in RPMI 1640 medium (Fujifilm Wako Pure Chemical Industries, Osaka, Japan) supplemented with 10% fetal bovine serum (FBS) at 37°C in 5% CO_2_. J774 cells stably expressing mVenus (mV), mV-MORN2, Myc, or Myc-SNAP-23 were maintained in RPMI supplemented with 10% FBS and 2 µM puromycin. J774/mV-MORN2 cells stably co-expressing Myc or Myc-SNAP-23 were cultured in RPMI with 10% FBS in the presence of 2 μM puromycin and 0.7 µM blasticidin. Phoenix-Ampho cells were maintained in DMEM (Sigma-Aldrich) supplemented with 10% FBS at 37°C in 5% CO_2_. Cell lines were recently authenticated and tested for contamination.

### Expression vectors and establishment of stable transfectants

MORN2 cDNA was obtained by reverse transcription PCR using total RNA extracted from J774 cells. The cDNA was cloned into the pmVenus-C1 and pFlag-CMV2 vectors and confirmed by DNA sequencing. mVenus-MORN2 cDNA was cloned into the pFlag-5a vector. pmVenus-MORN2 ΔC6 and pmVenus-MORN2 K/R were created by overlapping PCR and confirmed by DNA sequencing. Similarly, pmVenus-p62-HA was created from HA-p62 (Addgene plasmid #28027; Dr. Qing Zhong).

J774 cell lines stably expressing mV, mV-MORN2 wt, mV-MORN2 ΔC6, mV-MORN2 K/R, Myc, or Myc-SNAP-23 proteins were established by infection with recombinant retroviruses generated using cDNAs of mVenus-tagged and Myc-tagged proteins cloned into the pCXpur and pCXblast vector, respectively (Akagi et al., 2003).

### siRNA experiments

An siRNA duplex with 52% GC content (5′-GUACCGCACGUCAU­UCGUAUC-3′; Sigma-Aldrich) was used as a control. The RNA duplexes for mouse SNAP-23 and stx11 #1 were previously described (Sakurai et al., 2012; Kinoshita et al., 2019). J774/mV-MORN2, J774/mV-MORN2/Myc, and J774/mV-MORN2/Myc-SNAP-23 cells were transfected with control (20–50 nM final concentration), SNAP-23 siRNAs (20 nM), or syntaxin 11 siRNAs (50 nM) using the HiPerFect transfection reagent (Qiagen, Hilden, Germany) according to the manufacturer's instructions. The cells were used for subsequent experiments 3 days after transfection. The following RNA duplexes were used for targeting: MORN2 siRNA#1 (5′-GGAAAUUUCAAUGAAAAUATT-3′) and siRNA#2 (5′-CUAAAGCUCUACAUGUAGATT-3′) (JBioS, Saitama, Japan), corresponding to the open reading frame and 3′-untranslated region of mouse *MORN2* mRNA, respectively. J774 and J774/mV-MORN2 cells were transfected twice with control (100 nM final concentration) or MORN2 siRNAs (100 nM) as described above. The cells were used for subsequent experiments 4 days after transfection.

### WB and immunoprecipitation

Cell lysates were obtained with radio-immunoprecipitation assay (RIPA) buffer (25 mM Tris-HCl pH 7.2, 150 mM NaCl, 1% Triton X-100, 0.1% SDS, and 0.5% sodium deoxycholate) or extraction buffer (20 mM HEPES-KOH pH 7.2, 100 mM KCl, 2 mM EDTA, 1% Triton X-100, 1 mM dithiothreitol and a protease inhibitor cocktail [Nacalai Tesque, Kyoto, Japan]) and were treated at 95°C with 5× SDS-PAGE sample buffer. After SDS-PAGE, the samples were analyzed by WB using various antibodies. The immunoreactive proteins were visualized utilizing ImmunoStar Zeta (Fujifilm Wako Pure Chemical Industries) on the ImageQuant LAS-4000 system (GE Healthcare, Chicago, IL, USA). Protein signals were semi-quantified using ImageJ v.1.52a (National Institutes of Health, Bethesda, MD, USA).

Phoenix-Ampho cells transiently transfected with MORN2 constructs or J774/mVenus-tagged proteins were incubated in the presence or absence of MG132 (Cayman Chemical, Ann Arbor, MI, USA) at 5 µM final concentration for 5 h. Treatment with 5 µM MG132 did not affect cell viability (data not shown), which is consistent with the previously-reported finding in J774 cells (Alileche et al., 2006). The cell lysates were incubated with an anti-EGFP antibody for 30 min on ice. Next, protein A-Sepharose beads (GE Healthcare) were added and the mixture was incubated for 16 h at 4°C under gentle rotation. Subsequently, the beads were washed with RIPA buffer and the immune complexes were eluted with SDS-PAGE sample buffer. After SDS-PAGE, the samples were analyzed by WB using anti-ubiquitin and EGFP antibodies. Detection was performed as described above.

### Analysis of phagocytosis with Texas Red-conjugated zymosan or *E. coli*-mCherry-mVenus particles

J774/mV and J774/mV-MORN2 cells were subjected to phagocytosis analyses using Texas Red-conjugated zymosan particles (Fujifilm Wako Pure Chemical Industries) opsonized with or without IgG or *E. coli*-mCherry-mVenus particles as described previously (Hatsuzawa et al., 2009; Sakurai et al., 2012; Morita et al., 2017).

### Phagosome-lysosome fusion assay

J774/mVenus and J774/mVenus-MORN2 cells were plated onto the center of 35-mm-diameter glass-bottom dishes at a density of 0.75×10^6^ cells and labeled overnight at 37°C with 50 µg/ml CF640R-dextran (MW 10,000 Da; Biotium, Fremont, CA, USA), after which the labeling medium was removed. Subsequently, the cells were chased for 5 h, pre-incubated with or without 10 µM DPI for 30 min, washed with ice-cold Hank's balanced salt solution (HBSS) and incubated for 30 min on ice in the presence of an approximately 30-fold excess of Texas Red-conjugated zymosan. Next, the cells were maintained at 30°C for 5 min to initiate phagocytosis, washed with ice-cold HBSS, incubated with anti-zymosan antibodies for 20 min on ice and maintained with Alexa 405-conjugated anti-rabbit secondary antibodies to stain extracellular zymosan. Finally, the cells were incubated in HBSS-containing cytochalasin B (20 μM final concentration) for 15 min at 30°C. Images were captured on an LSM780 laser-scanning microscope with a Plan-Apochromat 63×/1.40 oil DIC M27 objec­tive lens (Carl-Zeiss, Oberkochen, Germany) under low temperature conditions (6°C). More than 40 phagosomes were counted for each experiment and categorized as CF640R-dextran-positive or unlabeled phagosomes based on the presence or absence of CF640R fluorescence signal.

### Analysis of LAP activity using *E. coli* or zymosan

J774/mV and J774/mV-MORN2 cells were incubated in the presence or absence of *E. coli* Rosetta (DE3) at an OD_600_ of approximately 1.0 unit for 1 h and were then used for subsequent experiments. WB was used to estimate the LAP efficiency by the density ratio of the generated LC3-II signal to the β-actin signal, and the ratio was normalized to the value obtained for cells without *E. coli* particles in the same experiment defined as 1.0.

J774/mVenus and J774/mV-MORN2 cells were incubated in the presence or absence of zymosan particles (100 μg/ml) opsonized with or without IgG for 60 min. The zymosan-containing medium was then replaced with fresh medium and the cells were further incubated for 30 min or 120 min. Cells labeled with anti-LC3 antibodies were used to analyze LC3-positive phagosomes (LAPosomes) as described in the following section.

J774/mV-MORN2, J774/mV-MORN2/Myc, and J774/mV-MORN2/Myc-SNAP-23 cells were pre-incubated with or without DPI (10 μM final concentration; Sigma-Aldrich) for 30 min. The cells were then incubated with zymosan particles in the presence or absence of DPI for 90 min. Cells stained with anti-LC3 antibodies were used to analyze LC3-positive phagosomes (LAPosomes) as described in the following section.

### Immunostaining

J774/mV-MORN2 cells were fixed with 100% methanol for 7 min at −20°C and incubated with 2% bovine serum albumin (BSA)/PBS for 30 min at 25°C. Subsequently, the cells were treated with anti-EGFP and anti-γ-tubulin antibod­ies and stained with Alexa 488-conjugated anti-rabbit IgG antibodies and Alexa 555-conjugated anti-mouse IgG antibodies.

For the LAPosome formation assay detecting LC3-positive phagosomes, the cells were fixed with 10% trichloroacetic acid for 10 min on ice and permeabilized with 0.2% Triton X-100/PBS for 5 min at 25°C. These treatments quenched almost all intracellular mVenus-derived signals. After blocking with Blocking One solution (Nacalai Tesque) for 30 min at 25°C, the cells were stained with anti-LC3 antibodies and with Alexa 488-conjugated anti-rabbit IgG antibodies.

J774/mVenus/Myc-SNAP-23 and J774/mV-MORN2/Myc-SNAP-23 cells were maintained in the presence of zymosan particles for 90 min. The cells were then fixed with 100% methanol for 7 min at −20°C and incubated with 2% BSA/PBS for 30 min at 25°C. Subsequently, the cells were stained with anti-Myc antibod­ies and Alexa 555-conjugated anti-mouse IgG antibodies to visualize phagosomes with a Myc fluorescent signal.

Images were obtained using an LSM780 laser-scanning microscope.

### Analysis of phagosomal ROS production using the Fc OxyBURST reagent

J774/mVenus/Myc-SNAP-23 and J774/mV-MORN2/Myc-SNAP-23 cells (at 0.5×10^6^ cells per well in a 24-well plate) were pre-incubated for 30 min at 37°C with or without DPI (final concentration 10 μM). To monitor ROS in phagosomes, the cells were cooled on ice, incubated with Fc OxyBURST green reagent (dichlorodihydrofluorescein [H_2_DCF]-conjugated bovine serum albumin [BSA] complexed with rabbit anti-BSA antibodies; Thermo Fisher Scientific) for 15 min on ice, and then further incubated in the presence or absence of DPI for 90 min at 37°C (Desai et al., 2007). After being thoroughly washed with PBS to remove the free reagent, cells were fixed with 4% paraformaldehyde/PBS for 15 min. Fluorescence from the Fc OxyBURST reagent was quantified in an Infinite F500 microplate reader (TECAN, Kawasaki, Japan) at excitation 485 nm/emission 535 nm. Subsequently, cells were permeabilized with 0.2% Triton X-100/PBS for 5 min at 25°C. After blocking with 2% BSA/PBS for 30 min at 25°C, cells were stained with Alexa 568-conjugated anti-rabbit IgG antibodies to assess the phagocytosis efficiency of the BSA immune complexes. Cellular fluorescence was quantified in the plate reader at excitation 535 nm/emission 612 nm. Arbitrary fluorescence units were obtained by subtraction of the fluorescence intensity observed in the absence of the Fc OxyBURST reagent from that observed in the presence of the reagent.

### Statistical analysis

Data are presented as mean±s.e.m. Intergroup differences were analyzed by a two-tailed, paired Student's *t*-test or by ANOVA with Tukey's post hoc test using GraphPad Prism (GraphPad Software, La Jolla, CA, USA). Statistical significance was defined as *P*<0.05.

## Supplementary Material

Supplementary information
