# Supplementary material for: Characterization of MORN2 stability and regulatory function in LC3-associated phagocytosis in macrophages
Source: Biol Open. 2020 Jun 23;9(6):bio051029. doi: 10.1242/bio.051029 (PMC7327995; doi:10.1242/bio.051029)
Supplement: Supplementary information [file biolopen-9-051029-s1.pdf]

Supplementary information

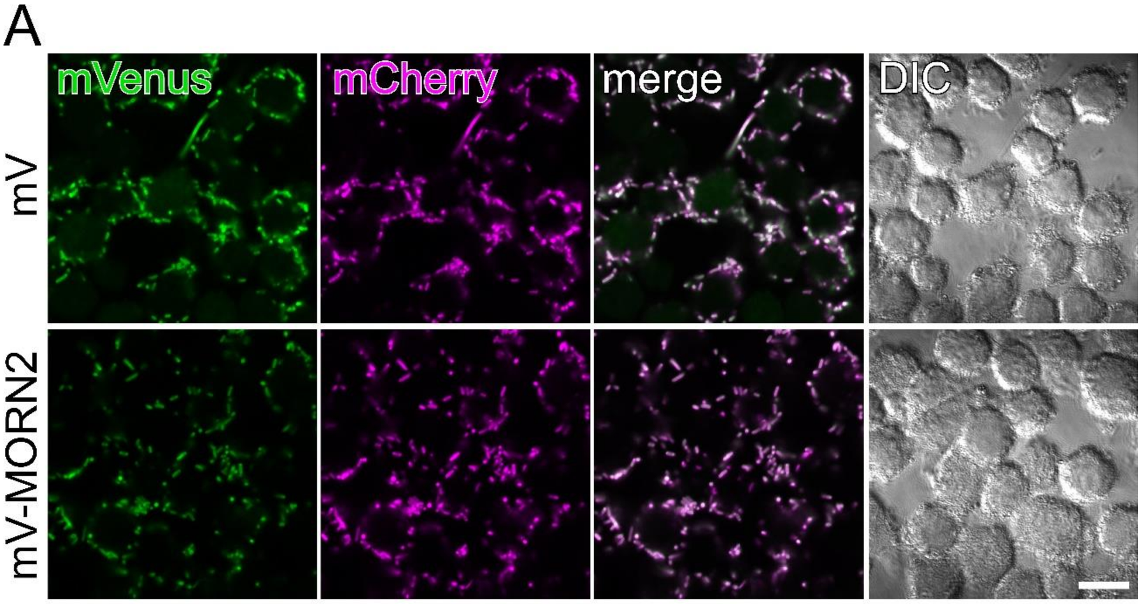

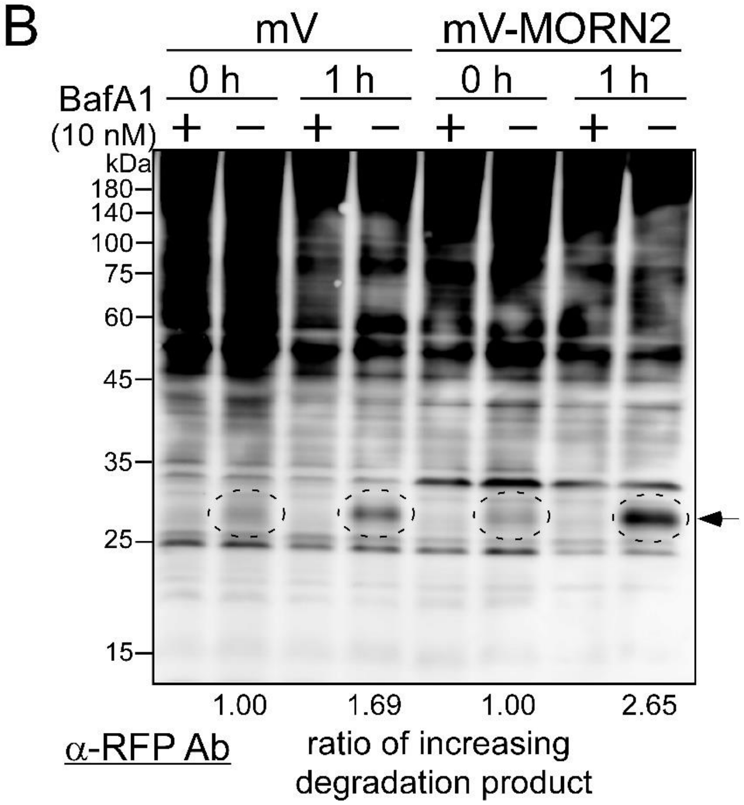

Fig. S1. J774/mVenus-MORN2 cells exhibit enhanced phagosomal acidification and degradation activity against ingested *E. coli*-mCherry-mVenus probes (related to Fig. 3). (A) After incubation

Biology Open • Supplementary information

with an *E. coli*-mCherry-mVenus probe for 1 h, PBS-washed mV or mV-MORN2 cells were fixed with 4% PFA/PBS at 4°C for 30 min. Images were captured with a confocal microscope. The fluorescent signal of mVenus and mVenus-MORN2 was substantially lower than that of the probe. Consistent with Fig. 3A, there was no difference in the phagocytosis efficiency (mCherry signal) between mV and mV-MORN2 cells. The mVenus signal of the *E. coli*-mCherry-mVenus probe in mV-MORN2 cells was relatively quenched compared with that in mV cells, which supports Fig. 3B. (B) Western blot analysis of *E. coli*-mCherry-mVenus probe degradation after ingestion by mV or mV-MORN2 cells in the presence or absence of 10 nM bafilomycin A1 (BafA1). Measurements at 0 h and 1 h represent samples taken immediately after 1 h incubation with the probe followed by washing to remove the unbound probe and again 1 h later, respectively. Whole-cell protein extracts were probed using an anti-RFP antibody (at 1:2000 dilution), which was a gift from Dr. Kenji Akasaki (Fukuyama University, Hiroshima, Japan) and Dr. Ikuo Wada (Fukushima Medical University, Fukushima, Japan). The arrow indicates the 28 kDa degradation product in each sample, and the dashed circle highlights the 28 kDa product in the absence of BafA1; BafA1 abolished the generation of this product. For each cell type, the level of the 28-kDa product observed at 0 h was defined as 1.00, and the density of the band after 1 h was normalized to this value (fold changes are indicated below the panel).

Biology Open • Supplementary information

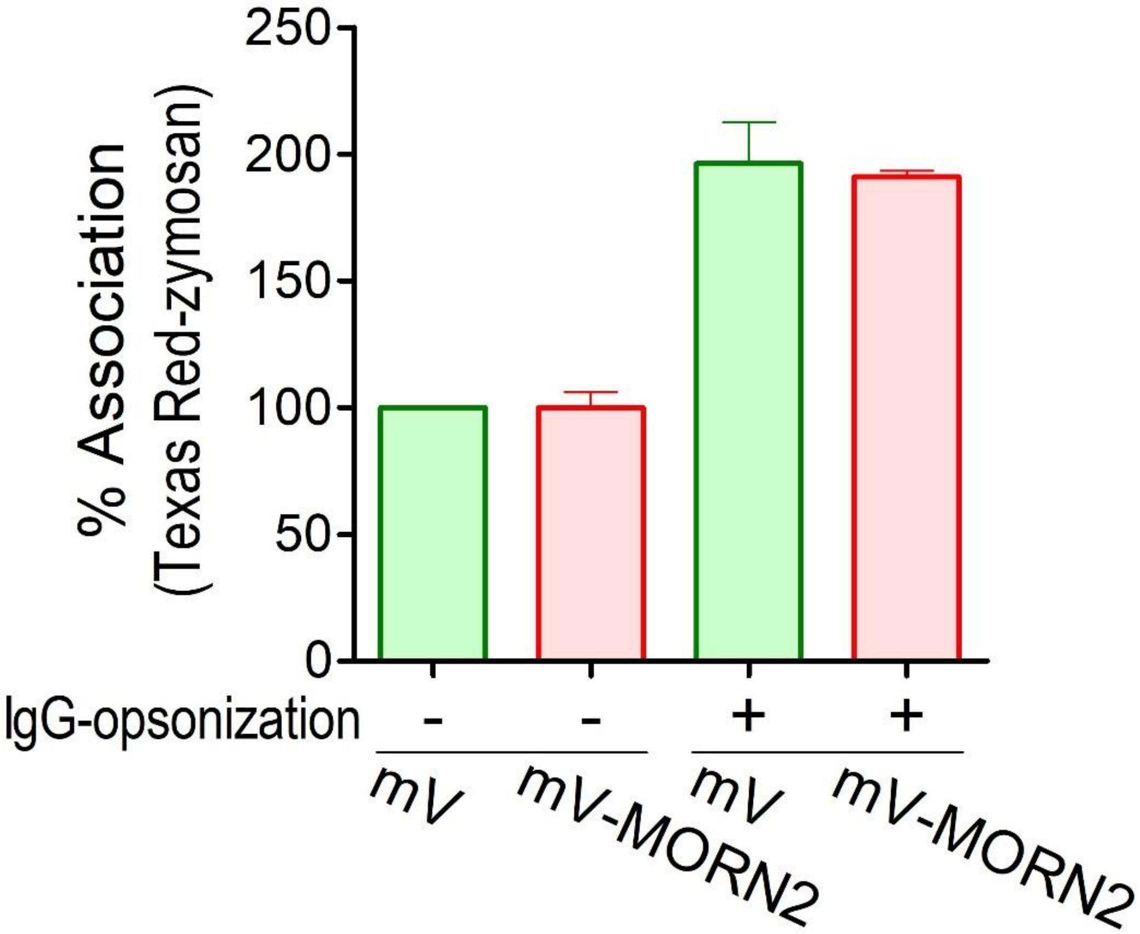

Fig. S2. There is no difference in the association efficiency of zymosan opsonized with or without IgG between cells expressing mV and mV-MORN2 (related to Fig. 4). J774 cells expressing mV-tagged proteins were incubated with Texas Red-zymosan particles opsonized with or without IgG, and the efficiencies (%) of the association were measured. Data are presented as mean ± SEM of three independent experiments.

Biology Open • Supplementary information

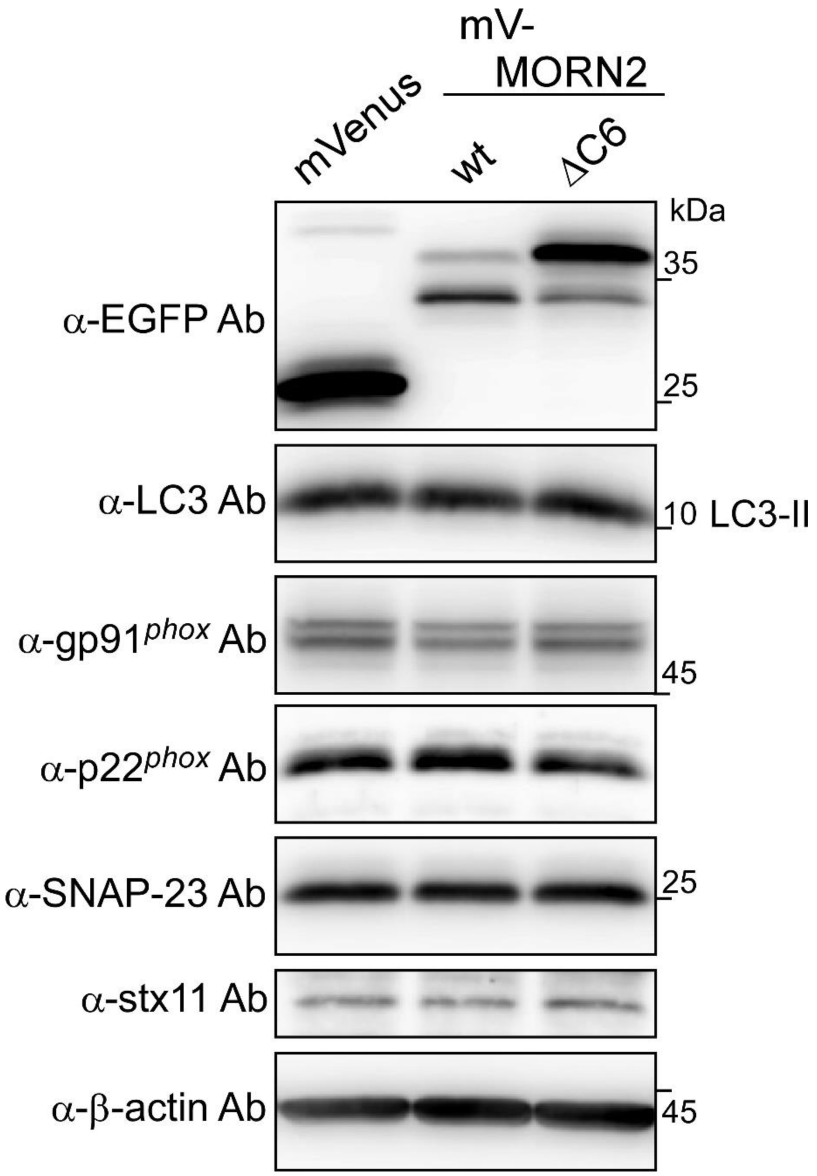

Fig. S3. Stable expression of mVenus-tagged proteins has no significant effect on the expression of other proteins in J774 cells (related to Fig. 4). Total protein lysates from J774 cells stably expressing mVenus, mV-MORN2 wild type (wt), or ΔC6 were analyzed by western blotting using the indicated antibodies.

Biology Open • Supplementary information

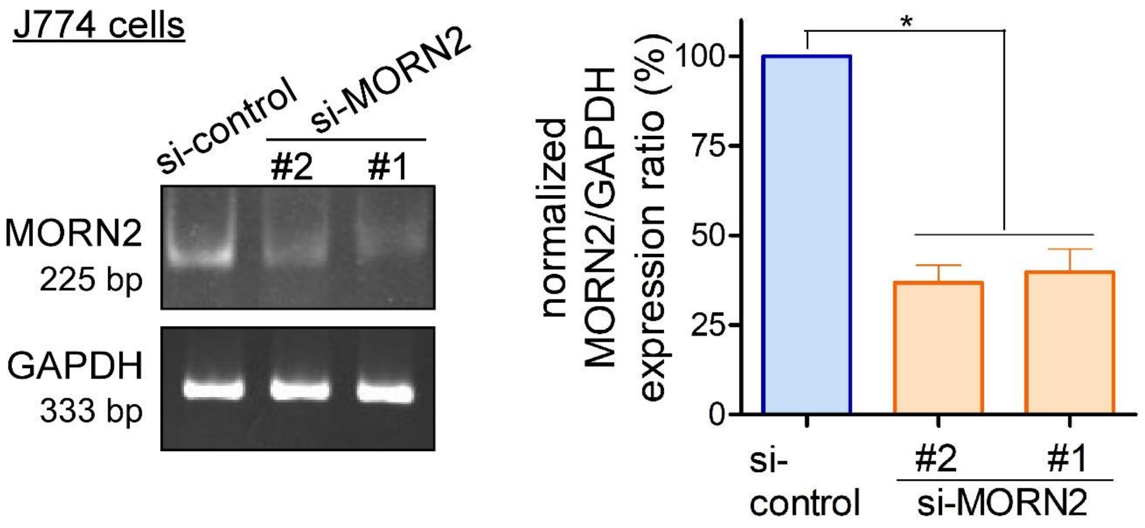

Fig. S4. Validation of the knockdown efficiency of siRNAs against mouse *MORN2* in J774 cells (related to Fig. 5). At 96 h after the transfection of siRNAs (100 nM each; see Materials and Methods) into J774 cells, total RNA was extracted by the RNeasy Mini Kit (Qiagen, Hilden, Germany) according to manufacturer's instructions. After incubation with DNase I (RNase-free; Takara, Kyoto, Japan) and an RNase inhibitor (Thermo Fisher Scientific, Waltham, MA) at 37°C for 30 min, total RNA samples (200–500 ng) were subjected to reverse transcription polymerase chain reaction (*RT-PCR*) using the PrimeScript II 1st Strand cDNA Synthesis Kit (Takara Bio, Kusatsu, Japan*)* according to manufacturer's instructions. Next, the *1st* strand cDNA was subjected to PCR using Tks Gflex DNA polymerase (Takara Bio) and KOD Plus (DNA polymerase; TOYOBO, Osaka, Japan) for *MORN2* (primer; 5′-TTCGAATTCGAATGGCTTTGGAAGACTTGA-3′ and 5′-GGTGGATCCTACATGTAGAGCTTTAGTTTC-3′) and *GAPDH* (primer; 5′-AAGGCTGTGGGCAAGGTCAT-3′ and 5′-CACCACCCTGTTGCTGTAGC-3′), respectively. PCR products of 255 bp for *MORN2* and 333 bp for *GAPDH* were detected on a 5% polyacrylamide gel (left panel) and quantified using ImageJ. The total value of the *MORN2* signal on the gel was expressed as a ratio of control siRNA and MORN2 siRNA#1 and #2 signals to

Biology Open • Supplementary information

each *GAPDH* signal and then normalized to the ratio of control siRNA signals, defined as 100%. Data are presented as mean ± SEM of three independent experiments. Statistical analysis was performed using a two-tailed, paired Student's *t*-test (right panel); $*p < 0.05$.

Biology Open • Supplementary information

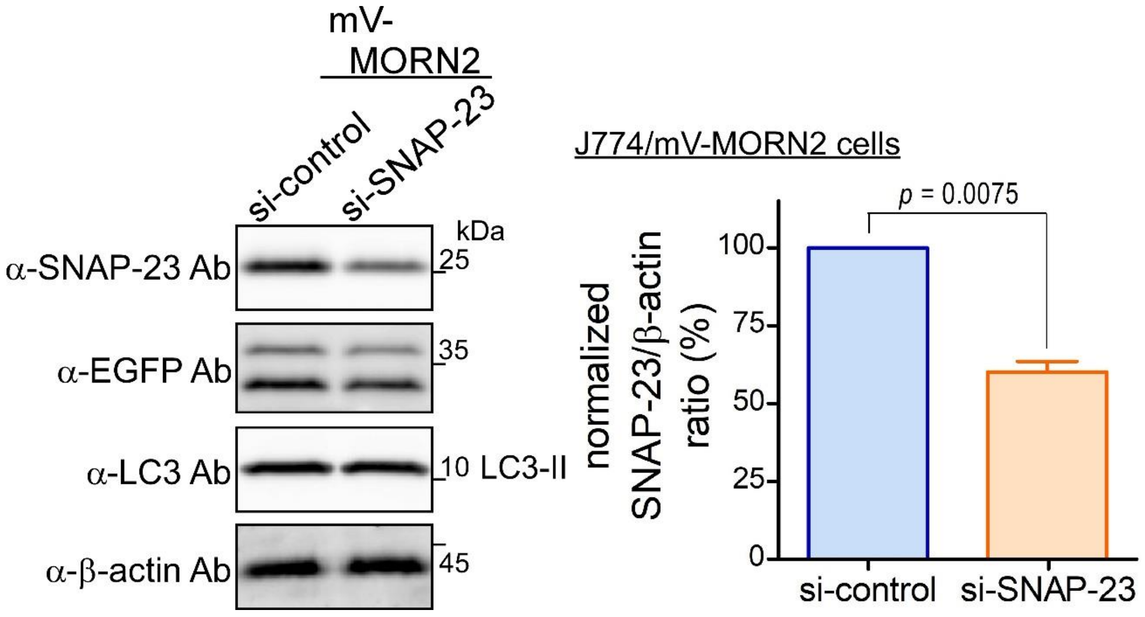

Fig. S5. Transfection of siRNA against mouse SNAP-23 reduces its protein expression without significantly affecting the expression of other proteins in J774/mV-MORN2 cells (related to Fig. 6). Total protein lysates from J774/mV-MORN2 cells at 72 h after transfection with SNAP-23 siRNA or control siRNA were analyzed by western blotting using the indicated antibodies (left panel). Protein signals were quantified utilizing ImageJ (right panel). The total value of SNAP-23 was expressed as a ratio of the protein signal in control and SNAP-23 siRNA cells to β-actin and then normalized to the ratio in control siRNA defined as 100%. Data are presented as mean ± SEM of three independent experiments. Statistical analysis was performed using a two-tailed, paired Student's *t*-test.

Biology Open • Supplementary information

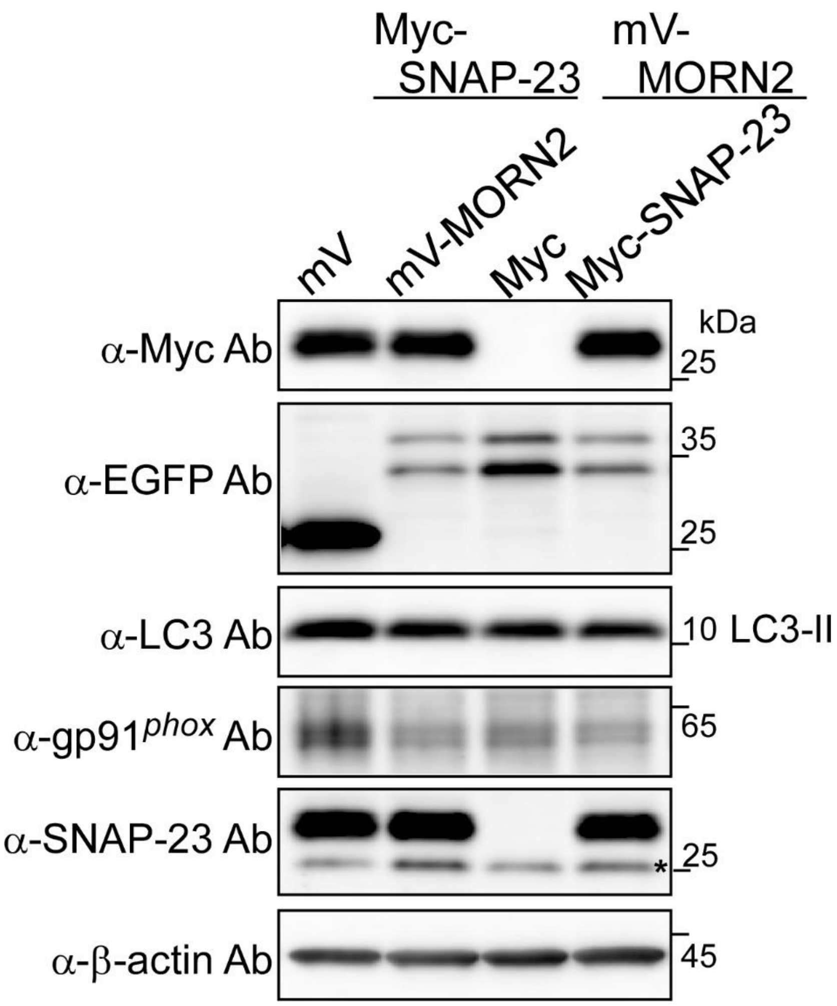

Fig. S6. Stable expression of both mVenus- and Myc-tagged proteins has no significant effect on the levels of other proteins in J774 cells (related to Fig. 6). Total protein lysates from J774 cells stably expressing both indicated combinations of mVenus-tagged and Myc-tagged proteins were analyzed by western blotting using the indicated antibodies. The asterisk indicates endogenous SNAP-23.

Biology Open • Supplementary information

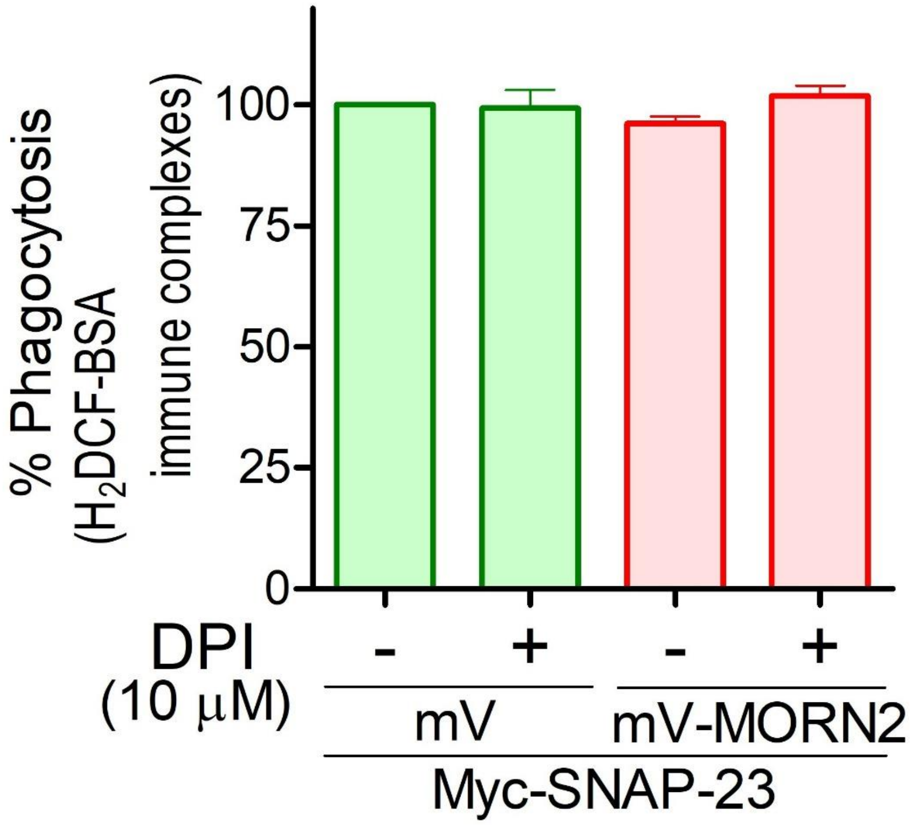

Fig. S7. There is no difference in the phagocytosis of the Fc OxyBURST reagent (H_2DCF-BSA immune complexes) between J774/Myc-SNAP-23 cells expressing mV and mV-MORN2 (related to Fig. 6). J774/Myc-SNAP-23 cells expressing mV-tagged proteins were incubated with the Fc OxyBURST reagent for 90 min in the presence or absence of DPI. After the measurement of the signal from the reagent (Fig. 6F), the cells were stained with Alexa 568-conjugated anti-rabbit IgG antibodies to visualize H_2DCF-BSA immune complexes in phagosomes, and then the efficiency (%) of phagocytosis was measured as described in the Materials and Methods. Data are presented as mean ± SEM of three independent experiments.

Biology Open • Supplementary information

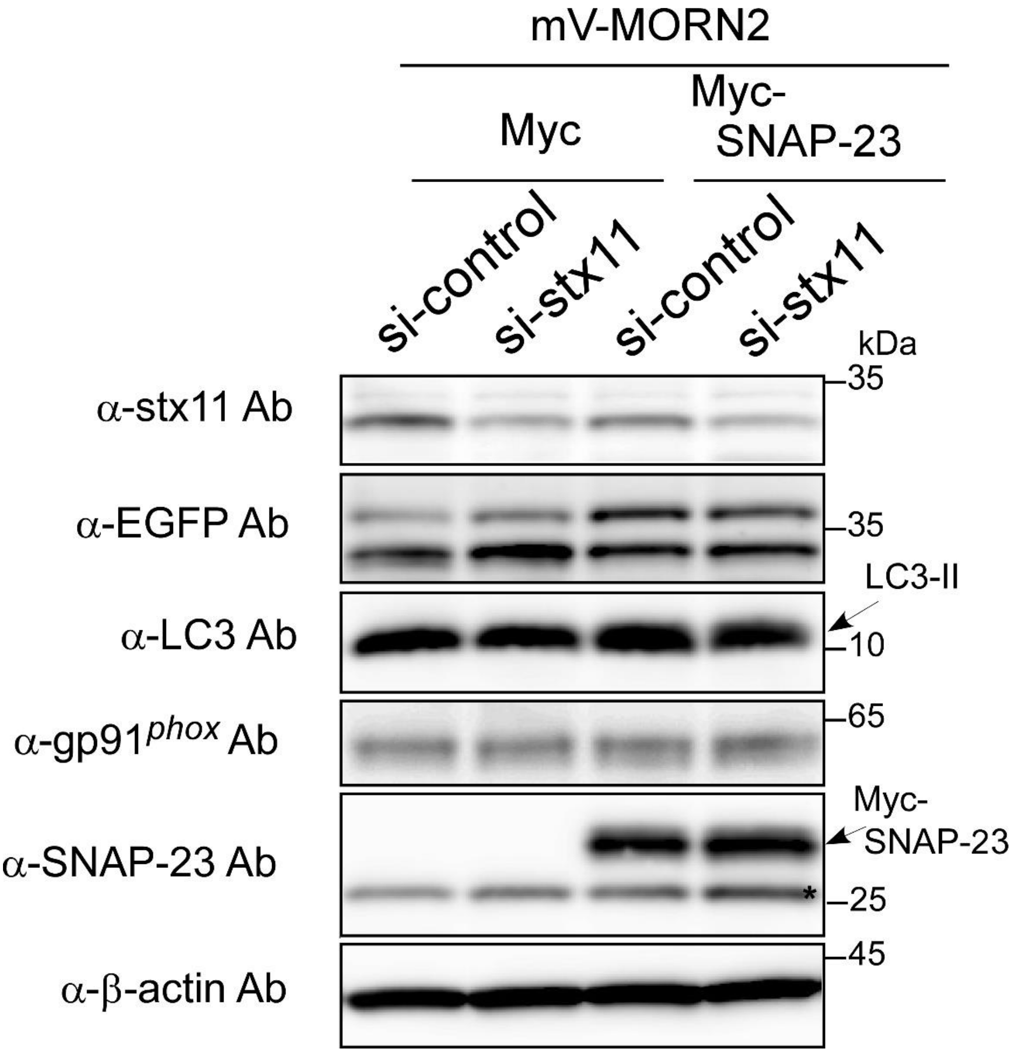

Fig. S8. Transfection of siRNA against mouse stx11 reduces its protein expression without significantly affecting the expression of other proteins in J774/mV-MORN2/Myc and J774/mV-MORN2/Myc-SNAP-23 cells (related to Fig. 7). Total lysates from J774/mV-MORN2/Myc and J774/mV-MORN2/Myc-SNAP-23 cells at 72 h after transfection with stx11 siRNA or control siRNA were analyzed by western blotting using the indicated antibodies.

Biology Open • Supplementary information
